# Bioinformatics Profiling of a Paeoniflorin-Associated “Macrophage–Lactylation” Axis in Hepatocellular Carcinoma: An LDHA-Based Prognostic Model and Structural Computational Evidence

**DOI:** 10.3390/ijms27052495

**Published:** 2026-03-09

**Authors:** Kongli Fan, Ruiqi Zhao, Jialing Sun, Jing Li, Minling Lv, Mengqing Ma, Jiesheng Guo, Xiaozhou Zhou

**Affiliations:** 1 The Fourth Clinical Medical College of Guangzhou University of Chinese Medicine, Shenzhen 518033, China; 2 Faculty of Chinese Medicine, Macau University of Science and Technology, Macao 999078, China

**Keywords:** hepatocellular carcinoma, paeoniflorin, lactylation, macrophage, prognostic model, molecular docking, molecular dynamics

## Abstract

Hepatocellular carcinoma (HCC) progression is shaped by crosstalk between the tumor immune microenvironment (TME) and metabolic reprogramming. This study aims to characterize a macrophage–lactylation molecular axis in HCC and to develop a quantitative prognostic stratification model. Using the TCGA-LIHC cohort, differentially expressed genes were intersected with Paeoniflorin (PF)-related targets, HCC disease targets, and macrophage-/lactylation-related genes to identify candidate genes. Prognostic genes were selected through Cox and LASSO-Cox analyses to construct a risk score model, followed by survival analysis and ROC curve evaluation. Immune infiltration was assessed using ESTIMATE and ssGSEA algorithms, and PF–protein binding interactions were explored via molecular docking and molecular dynamics simulations. Intersection analysis identified eight key genes, and prognostic model genes (*HNRNPU*, *LDHA*, and *NPM1*) were used to construct the prognostic model. High-risk patients exhibited significantly poorer overall survival (*p* < 0.001), with 1- and 3-year AUC values ranging from 0.70 to 0.90. *HNRNPU* was positively correlated with activated CD4 T cells (r = 0.385) and negatively correlated with eosinophils (r = −0.498). Molecular docking indicated favorable binding of PF to the model proteins, with the highest predicted affinity observed for LDHA (Vina score = −8.9 kcal/mol), and molecular dynamics simulations suggested the formation of a stable LDHA–PF complex during the later stage of the simulation. We propose a prognostic risk model for HCC constructed using three prognostic model genes and provide computational evidence linking PF to key molecular nodes such as LDHA. External cohort validation and experimental studies are warranted.

## 1. Introduction

Hepatocellular carcinoma (HCC) is the sixth most common cancer worldwide and the third leading cause of cancer-related death. According to GLOBOCAN 2022 estimates, there were approximately 870,000 new liver cancer cases and 760,000 deaths worldwide, ranking sixth in incidence and third in cancer-related mortality, and in China, the disease burden is particularly heavy, with an estimated 370,000 new cases and 320,000 deaths annually, representing nearly half of the global burden. Although targeted therapies and immunotherapies have advanced substantially, heterogeneous responses and treatment resistance continue to highlight the need for interpretable and actionable therapeutic targets and strategies [1,2]. In recent years, lactate accumulation driven by tumor metabolic reprogramming has been recognized not only as a metabolic hallmark reflecting energetic demand, but also as a signaling intermediate that could convey metabolic cues to transcriptional regulation through lactylation, thereby shaping tumor biology and the immune microenvironment. Meanwhile, macrophages are a major component of the tumor immune microenvironment, and their functional polarization is closely linked to metabolic signals. However, in HCC, the “lactylation–macrophage” axis and its druggable potential remain insufficiently characterized [3].

Paeoniflorin (PF) is a pharmacologically active natural product with reported anti-inflammatory and immunomodulatory effects [4]. In addition, recent studies focusing on immune cell functional reprogramming have suggested that PF might act on metabolism-related targets (e.g., glycolysis-associated proteins) and modulate microglia/macrophage polarization, thereby providing supportive evidence for a pharmacological narrative centered on “metabolism–immunity coupling” [5]. Nevertheless, the targetable molecular network and key binding targets of PF in HCC remain unclear. From a drug-development perspective, clarifying the core targets, relevant pathways, and their links to tumor immunity and metabolism might inform natural-product repositioning and combination strategies.

Therefore, we aimed to construct and validate a core-gene prognostic risk model for HCC by integrating TCGA-LIHC transcriptomic data. This model was developed through intersection analysis of PF targets, HCC disease targets, and macrophage-/lactylation-related genes. We further explore the clinical relevance, immune associations, and druggability of the PF-related axis, employing immune infiltration analyses, molecular docking, and molecular dynamics simulations to evaluate binding stability.

## 2. Results

### 2.1. Workflow

**Figure 1:**
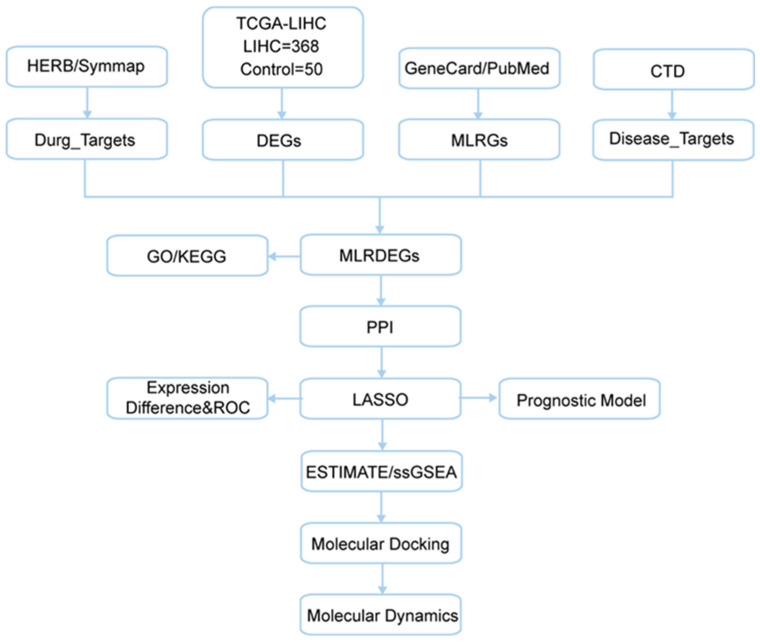
TCGA, The Cancer Genome Atlas; LIHC, Liver Hepatocellular Carcinoma; DEGs, differentially expressed genes; MLRGs, macrophage- and lactylation-related genes; MLRDEGs, macrophage and lactyling-related differentially expressed genes; CTD, Comparative Toxicogenomics Database; GO, Gene Ontology; KEGG, Kyoto Encyclopedia of Genes and Genomes; LASSO, Least Absolute Shrinkage and Selection Operator; ROC, Receiver Operating Characteristic; ESTIMATE, Estimation of Stromal and Immune cells in Malignant Tumor tissues using Expression; ssGSEA, Single-Sample Gene Set Enrichment Analysis.

The overall workflow of this study is summarized in Figure 1.

### 2.2. Identification of HCC- and Paeoniflorin-Related Macrophage- and Lactylation-Associated DEGs

**Figure 2:**
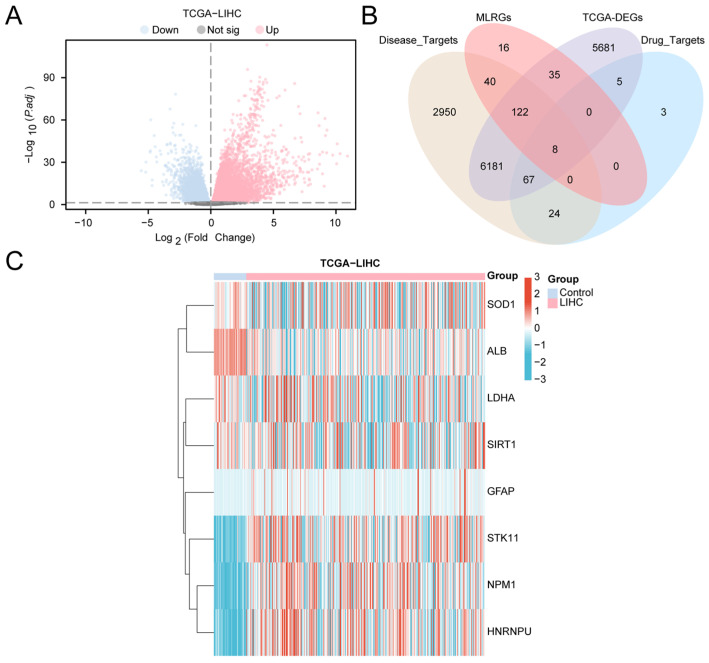
Differential Gene Expression Analysis. (**A**) Volcano plot of differentially expressed genes analysis between liver cancer (LIHC) group and control (Control) group in the liver cancer dataset (TCGA-LIHC). (**B**) Venn diagram of differentially expressed genes (DEGs), Drug_Targets, Disease_Targets and MLRGs in the liver cancer dataset (TCGA-LIHC). (**C**) Heat map of MLRDEGs in the liver cancer dataset (TCGA-LIHC). MLRDEGs; DEGs, Differentially Expressed Genes. Pink is the liver cancer (LIHC) group and light blue is the control (Control) group. In the heat map, red represents high expression and blue represents low expression.

Based on the TCGA-LIHC cohort, samples were stratified into the LIHC (tumor) group and the control group, and differential expression analysis was performed using DESeq2. In total, 12,099 significantly differentially expressed genes (DEGs; |logFC| > 0, adjusted *p* < 0.05) were identified, including 8130 upregulated and 3969 downregulated genes. The results are visualized in a volcano plot (Figure 2A), indicating extensive transcriptomic reprogramming in HCC tissues.

To further prioritize molecules potentially linking paeoniflorin intervention to HCC progression and to macrophage- and lactylation-related regulation, we intersected DEGs with paeoniflorin targets, HCC disease targets, and macrophage- and lactylation-related genes (MLRGs). The four-way overlap yielded eight macrophage- and lactylation-related differentially expressed genes (MLRDEGs), illustrated by a Venn diagram (Figure 2B; gene list in Table S4). These MLRDEGs represent a convergent set positioned at the intersection of drug relevance, disease association, and immunometabolic regulation.

We next visualized the expression patterns of the eight MLRDEGs across LIHC and control samples using a heatmap (Figure 2C). All MLRDEGs showed clear differential expression between groups, suggesting potential involvement in HCC biology and pathways that might be modulated by paeoniflorin.

### 2.3. Gene Ontology and Kyoto Encyclopedia of Genes and Genomes Enrichment Analysis

**Figure 3:**
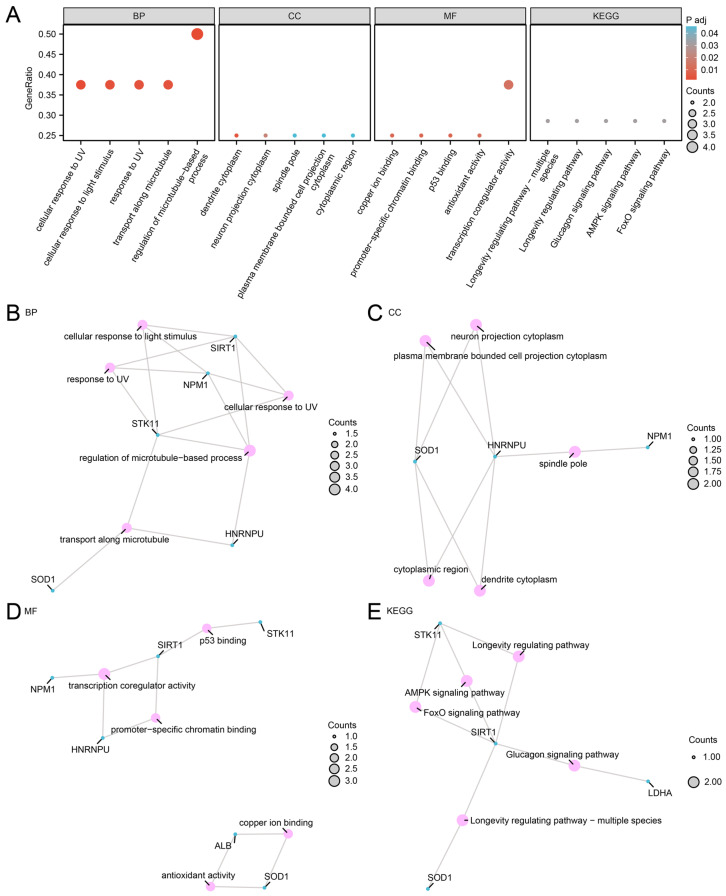
GO and KEGG Enrichment Analysis for MLRDEGs. (**A**) Bubble plot of GO and KEGG enrichment analysis results of MLRDEGs: biological process (BP), cellular component (CC), molecular function (MF) and biological pathway (KEGG). GO terms and KEGG terms are shown on the abscissa. (**B**–**E**) GO and KEGG enrichment analysis results of MLRDEGs network diagram showing BP (**B**), CC (**C**), MF (**D**) and KEGG (**E**). Pink nodes represent items, blue nodes represent molecules, and lines represent the relationship between items and molecules. MLRDEGs; GO; KEGG; BP, Biological Process; CC; MF. The bubble size in the bubble plot represents the number of genes, and the color of the bubble represents the size of the adjusted *p*-value, the reder the color, the smaller the adjusted *p*-value, and the bluer the color, the larger the adjusted *p*-value. The screening criteria for GO and KEGG enrichment analysis were adj. *p* < 0.05 and FDR value (q value) < 0.25, and the *p*-value correction method was Benjamini–Hochberg (BH).

To characterize the functional landscape of the eight MLRDEGs, GO (Gene Ontology) and KEGG (Kyoto Encyclopedia of Genes and Genomes) enrichment analyses were conducted (Table 1). GO biological process (BP) terms were primarily enriched in the regulation of microtubule-based processes, cellular responses to UV and light stimuli, and transport along microtubules. In the cellular component (CC) category, enrichment was observed in dendrite cytoplasm, neuron projection cytoplasm, spindle pole, and plasma membrane-bounded cell projection cytoplasm. Molecular function (MF) terms included transcription coregulator activity, copper ion binding, promoter-specific chromatin binding, p53 binding, and antioxidant activity. KEGG analysis further suggested enrichment in longevity-regulating pathways, glucagon signaling pathways, AMPK signaling pathways, and FoxO signaling pathways. Enrichment results are presented as a bubble plot (Figure 3A) and as BP/CC/MF/KEGG network visualizations (Figure 3B–E) to illustrate gene–term relationships.

### 2.4. PPI Network and Functional Extension

**Figure 4:**
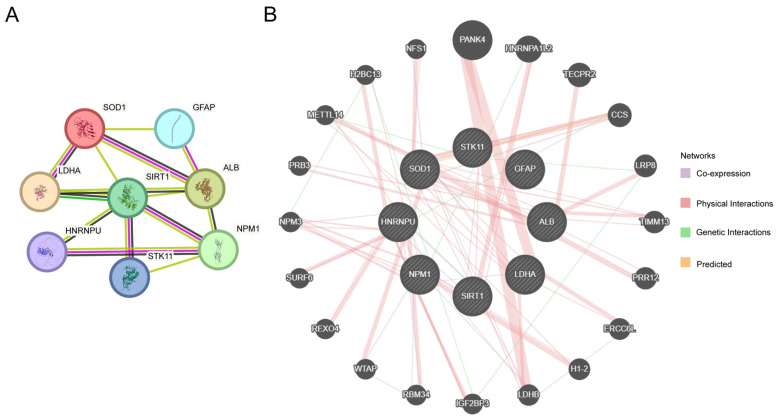
PPI Network Analysis. (**A**) PPI network of MLRDEGs calculated by STRING database. (**B**) Interaction network of MLRDEGs and genes with similar functions predicted by GeneMANIA website. The circles in the figure represent MLRDEGs and genes with similar functions, and the colors of the lines represent different interrelated functions.

To explore potential functional connectivity among the eight MLRDEGs, a protein–protein interaction (PPI) network was constructed using STRING (Figure 4A). Interactions of varying degrees were observed among SOD1, LDHA, ALB, NPM1, SIRT1, GFAP, STK11, and HNRNPU, implying that these genes might participate in processes related to metabolic regulation, stress responses, and immune–microenvironmental remodeling in HCC.

GeneMANIA was further used to extend functional associations, generating an expanded network comprising the eight MLRDEGs and 20 functionally related genes (Figure 4B). The diverse edge types (e.g., co-expression, shared protein domains, and predicted functional similarity) provide additional support for the potential coordinated roles of these genes in metabolism, stress adaptation, RNA processing, and immune-related functions, thereby informing downstream prioritization and model development.

### 2.5. Construction of a Prognostic Risk Model in HCC

**Figure 5:**
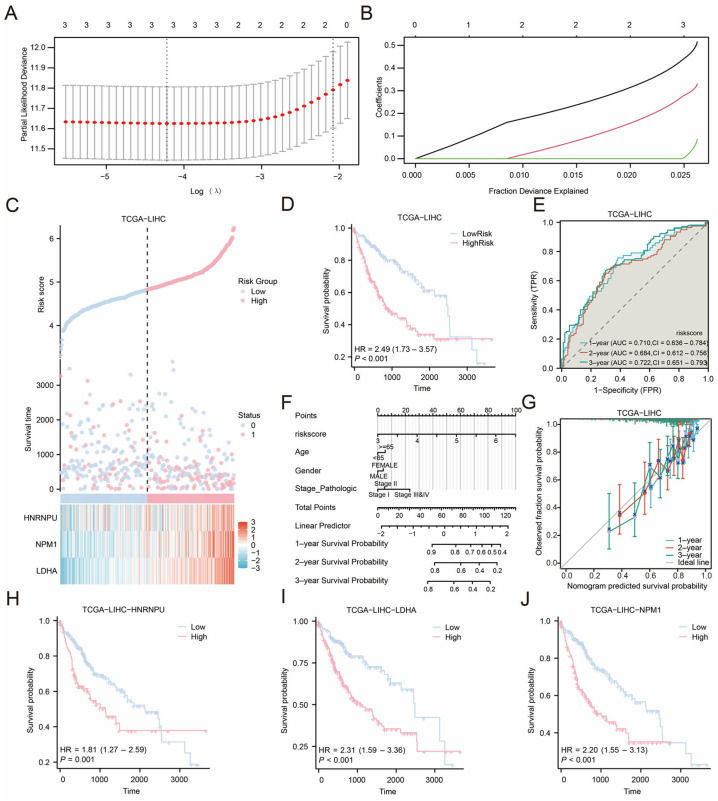
LASSO Regression Analysis. (**A**,**B**) Plots of prognostic risk models (**A**) and variable trajectories (**B**) from the LASSO regression model. (**C**) Risk factor plot of prognostic model genes prognostic LASSO model. (**D**) Prognostic KM curves between high and low risk score groups of LASSO and overall survival (OS) in the LIHC group. (**E**) Time-dependent ROC curves of the liver cancer (LIHC) group in the liver cancer dataset (TCGA-LIHC). The ROC curve located above the gray diagonal line means that the model has some discriminatory power and can effectively distinguish between positive and negative classes. (**F**) LASSO risk score and nomogram of clinical information in hepatocellular carcinoma (LIHC). (**G**) 1, 2, and 3-year Calibration curves of the prognostic risk model for liver cancer (LIHC) in the liver cancer dataset (TCGA-LIHC). (**H**,**J**) Prognostic KM curve between the expression of prognostic model genes (*HNRNPU* (**H**), *LDHA* (**I**), *NPM1* (**J**)) and the overall survival (OS) of HCC patients. LIHC, Liver Hepatocellular Carcinoma; MLRDEGs, macrophage- and lactylation-related differentially expressed genes; LASSO, Least Absolute Shrinkage and Selection Operator; OS, Overall Survival; KM, Kaplan–Meier; ROC, Receiver Operating Characteristic Curve; AUC, Area Under the Curve; TPR, True Positive Rate; FPR, False Positive Rate; TCGA. When AUC > 0.5, it indicates that the expression of the molecule is a trend to promote the occurrence of the event, and the closer the AUC is to 1, the better the diagnostic effect. AUC between 0.5 and 0.7 had low accuracy.

To evaluate the prognostic utility of MLRDEGs in HCC, univariate Cox regression was performed in TCGA-LIHC tumor samples, and genes with *p* < 0.10 were entered into a LASSO-Cox model. Based on the LASSO coefficient profiles and cross-validation curve (Figure 5A,B), three genes with the strongest prognostic contribution were retained—*HNRNPU*, *LDHA*, and *NPM1*—and were defined as the prognostic model genes. A risk score formula was subsequently constructed accordingly.

Risk score distributions and survival status are shown in Figure 5C, where a higher event rate was observed in the high-risk group. Kaplan–Meier analysis using the median risk score as the cutoff demonstrated significantly poorer overall survival in the high-risk group (Figure 5D, *p* < 0.001). Time-dependent ROC curves (Figure 5E) indicated that the AUCs at 1 and 3 years ranged from 0.7 to 0.9, suggesting acceptable discrimination in this cohort. To facilitate clinical interpretability, a nomogram incorporating the risk score and key clinical variables was constructed (Figure 5F), in which the risk score contributed most prominently. Calibration curves at 1, 2, and 3 years (Figure 5G) suggested the best agreement at 3 years for survival prediction. Finally, Kaplan–Meier analyses for each prognostic model gene (Figure 5H–J) showed that the expression levels of *HNRNPU*, *LDHA*, and *NPM1* were significantly associated with survival (*p* < 0.05), supporting their potential relevance as prognostic markers for further investigation.

### 2.6. Differential Expression Validation and ROC Analysis of Prognostic Model Genes

**Figure 6:**
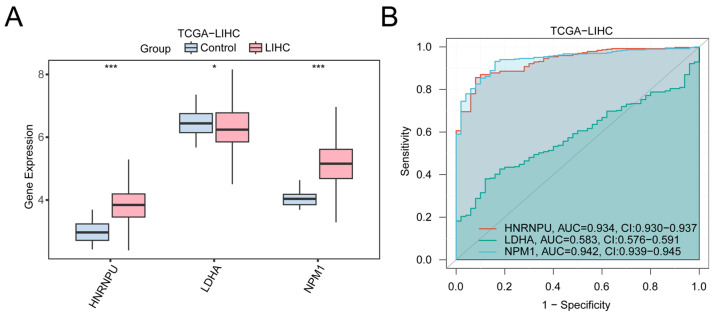
Differential expression validation and ROC curve analysis of prognostic model genes. (**A**) Group comparison diagram of prognostic model genes in liver cancer (LIHC) samples and control samples of the liver cancer dataset (TCGA-LIHC). (**B**) Prognostic model genes: ROC curves of *HNRNPU*, *LDHA* and *NPM1* in the liver cancer dataset (TCGA-LIHC). * represents *p* < 0.05, indicating statistical significance; *** represents *p* < 0.001, highly statistically significant. When AUC > 0.5, it indicates that the expression of the molecule is a trend to promote the occurrence of the event, and the closer the AUC is to 1, the better the diagnostic effect. AUC values between 0.5 and 0.7 were associated with lower accuracy, and AUC values greater than 0.9 were associated with higher accuracy. TCGA; LIHC, Liver Hepatocellular Carcinoma; ROC, Receiver Operating Characteristic; AUC, Area Under the Curve. In the group comparison plot, pink represents the liver cancer (LIHC) group and light blue represents the control (Control) group. The ROC curve located above the gray diagonal line means that the model has some discriminatory power and can effectively distinguish between positive and negative classes.

To assess the expression characteristics and diagnostic potential of the prognostic model genes in LIHC, we compared the expression levels of *HNRNPU*, *LDHA*, and *NPM1* between tumor and normal tissues (Figure 6A). *HNRNPU* and *NPM1* were significantly upregulated in LIHC (*p* < 0.001), whereas *LDHA* also exhibited an increasing trend.

ROC analyses based on gene expression (Figure 6B) indicated AUC values greater than 0.90 for *HNRNPU* and *NPM1*, whereas *LDHA* exhibited a more modest AUC (0.5–0.7), implying limited standalone diagnostic performance. Nevertheless, within the context of the multi-gene prognostic model, these genes might collectively contribute to risk stratification.

### 2.7. Association Between Risk Model and Tumor Immune Microenvironment in LIHC

**Figure 7:**
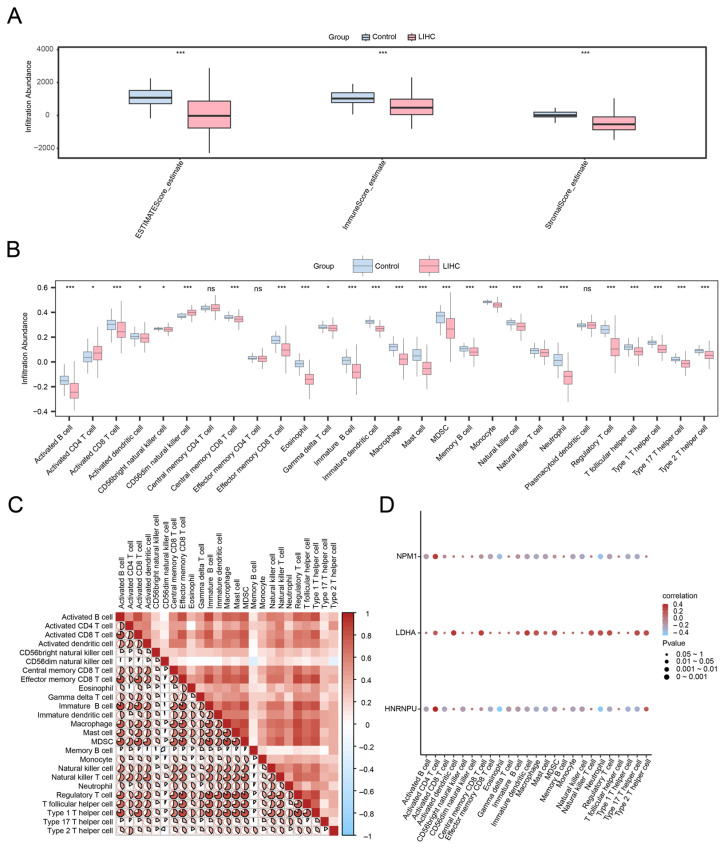
ESTIMATE and immune infiltration analysis by ssGSEA algorithm. (**A**) Group comparison plot of immunoscore results of ESTIMATE analysis between liver cancer (LIHC) and control (Control) groups of the liver cancer dataset (TCGA-LIHC). Group comparison plot of (**B**) immune cells in liver cancer (LIHC) and control (Control) in the liver cancer dataset (TCGA-LIHC). (**C**) Correlation heat map of immune cell infiltration abundance in the liver cancer dataset (TCGA-LIHC). The proportion and color intensity of the pie chart represent the strength of the correlation. The transition from red to blue on the color bar represents the change from high to low correlation. (**D**) Bubble plot of correlation between prognostic model genes and immune cell infiltration abundance in the liver cancer dataset (TCGA-LIHC). ssGSEA; TCGA; LIHC, Liver Hepatocellular Carcinoma; ns stands for *p*-value ≥ 0.05, not statistically significant; * represents *p*-value < 0.05, statistically significant; ** represents *p*-value < 0.01, highly statistically significant; *** represents *p*-value < 0.001 and highly statistically significant. Red indicates a positive correlation and blue indicates a negative correlation. The depth of the color represents the strength of the correlation. Pink represents the liver cancer (LIHC) group and light blue represents the control (Control) group.

To investigate the relationships between the risk model and the tumor immune microenvironment (TME), ESTIMATE (Estimation of Stromal and Immune cells in Malignant Tumor tissues using Expression)-derived stromal, immune, and composite ESTIMATE scores were calculated. Compared with normal tissues, LIHC samples exhibited significantly reduced stromal and immune scores (Figure 7A, *p* < 0.001), suggesting an overall attenuation of immune and stromal components in tumor tissues.

Using ssGSEA to quantify the infiltration of 28 immune cell types, 25 immune cell categories differed significantly between tumor and control groups (Figure 7B). Many immune cell types showed strong positive correlations with one another (Figure 7C), consistent with coordinated immune regulation within the TME. Correlation analysis between the prognostic model genes and immune infiltration (Figure 7D) revealed the strongest positive association between *HNRNPU* and activated CD4 T cells (r = 0.385, *p* < 0.05), as well as the strongest negative association between *HNRNPU* and eosinophils (r = −0.498, *p* < 0.05), although these coefficients represent weak-to-moderate correlations. These findings suggest that the prognostic model genes might be linked to the immune contexture in HCC and provide hypotheses for subsequent mechanistic validation.

### 2.8. Exploratory Molecular Docking and Molecular Dynamics Analyses of Paeoniflorin with Model Gene-Encoded Proteins

**Figure 8:**
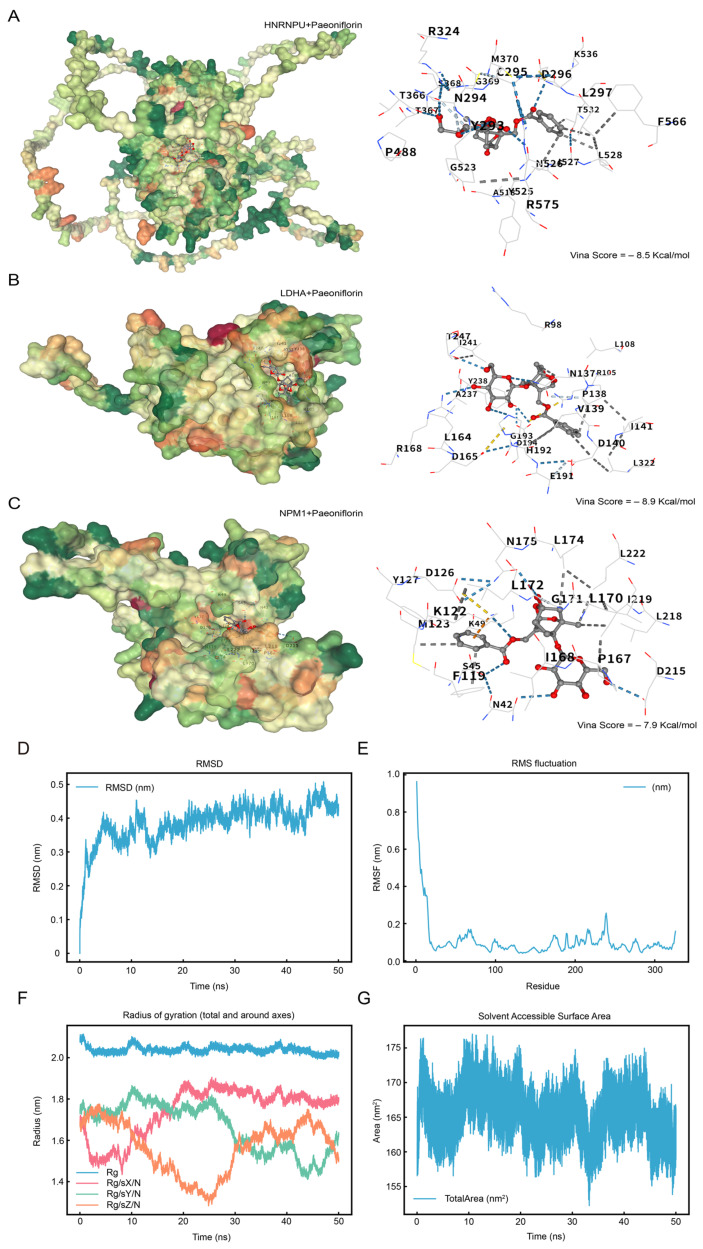
Molecular Docking. (**A**–**C**). Prognostic model genes *HNRNPU* and Paeoniflorin (**A**), Prognostic model genes *LDHA* and Paeoniflorin (**B**), Prognostic model genes *NPM1* and Paeoniflorin (**C**), the docking results are visualized, from left to right, respectively, the docking global map and the interaction force map. The surface color changes from green, orange, and red, indicating the change in amino acid property from hydrophilic to hydrophobic. Blue dashed lines—hydrogen bonds, light blue dashed lines—weak hydrogen bonds, gray dashed lines—hydrophobic forces, and orange dashed lines—cation-π interactions. Molecular Dynamics Simulation. (**D**–**G**). Molecular Dynamics Simulation. (**D**). Plot of the root mean square deviation (RMSD) as a function of time, with the X-axis showing the time (in nanoseconds, ns) and the Y-axis showing the RMSD value (in nanometer, nm). (**E**). RMSF (RMSF) plot, with atom numbers on the X-axis and RMSF values (in nanometer, nm) on the Y-axis. (**F**). Plot of gyration radius (Rg) as a function of time, with X-axis showing time (in nanoseconds, ns) and Y-axis showing gyration radius (in nanometers, nm). (**G**). The change in solvent accessible surface area (SASA) over time, the X-axis shows the time (in nanoseconds, ns), and the Y-axis shows the SASA value (in square nanometers, nm^2^). RMSD, Root Mean Square Deviation; RMSF, Root Mean Square Fluctuation; Rg, Radius of Gyration; SASA, Solvent Accessible Surface Area.

To explore potential interactions between paeoniflorin and the proteins encoded by the prognostic model genes, molecular docking was performed for HNRNPU, LDHA, and *NPM1* (Figure 8). Paeoniflorin exhibited favorable docking to all three proteins, with the lowest predicted binding energy observed for the LDHA–paeoniflorin complex (Vina score = −8.9 kcal/mol), suggesting the strongest predicted affinity among the tested targets.

The interaction network included hydrogen bonds, hydrophobic contacts, and weak hydrogen bonds, which together might contribute to complex stability and potentially influence catalytic activity or conformational dynamics. These results provide in silico structural evidence supporting the plausibility of paeoniflorin–target engagement and informing subsequent experimental prioritization.

To further assess the reliability of the docking pose, a 50 ns molecular dynamics (MD) simulation was conducted for the docking conformation with the lowest binding energy, namely the paeoniflorin–*LDHA* complex. Structural stability and dynamic behavior were evaluated using RMSD, RMSF, radius of gyration (Rg), and solvent-accessible surface area (SASA) (Figure 8D–G).

RMSD analysis (Figure 8D) showed that, after an initial relaxation during the first 10 ns, the backbone RMSD converged and remained stable at approximately 0.15 nm for the remainder of the simulation, indicating an equilibrated complex suitable for downstream interpretation. RMSF analysis (Figure 8E) suggests that higher fluctuations were primarily localized to loop regions and terminal residues, whereas residues near the binding pocket exhibited relatively lower flexibility, consistent with a stabilizing effect of ligand binding. The Rg trajectory (Figure 8F) fluctuated within a narrow range, implying no major expansion or contraction of the protein fold. SASA values (Figure 8G) remained stable at 155–175 nm^2^, without abrupt exposure or collapse events. Collectively, the MD results support a structurally stable paeoniflorin–*LDHA* complex under simulated physiological conditions, lending additional confidence to the predicted binding mode and motivating further biochemical and cellular validation.

## 3. Discussion

HCC progression is driven not only by genetic alterations but also by a profoundly immunosuppressive TME [6], in which TAMs and metabolic reprogramming—particularly lactate accumulation—play pivotal roles in suppressing antitumor immunity. M2-like polarization of TAMs, partially induced by metabolic cues, contributes to therapeutic resistance and unfavorable clinical outcomes, thereby underscoring the need for integrative strategies that concurrently target immune regulation and metabolic signaling [7,8,9].

The core value of this study lies in the integration of public transcriptomic datasets, immune-infiltration association analyses, and molecular docking and MD simulations to propose a testable mechanistic hypothesis and to develop a prognostic model that may facilitate risk stratification. Importantly, this work represents a hypothesis-generating paradigm: rather than demonstrating a causal in vivo or in vitro effect of paeoniflorin, we leverage convergent multi-modal evidence to connect a “candidate target–immunometabolic circuitry–prognostic association” into an experimentally tractable framework, thereby prioritizing high-value targets and directions for subsequent mechanistic validation and pharmacological evaluation. Within this framework, we identified and established a three-gene prognostic signature comprising *HNRNPU*, *LDHA*, and *NPM1*. The model exhibited good discriminative capacity and favorable time-dependent predictive performance in the discovery cohort, supporting its potential utility for patient risk stratification. More importantly, these three prognostic model genes are not merely statistical features; they can be positioned within a unified immunometabolic mechanistic landscape. To date, no studies have investigated the roles of *HNRNPU* and *NPM1* within this immunometabolic framework. Thus, we propose a novel hypothesis that paeoniflorin may attenuate LDHA-dependent lactate production and lactate signaling, thereby modulating lactate/lactylation-driven TAM polarization and immunosuppressive programs, while potentially concurrently interfacing with HNRNPU- and NPM1-linked transcriptional regulation and immune-evasion circuits. Collectively, this integrated axis may reshape the HCC immune microenvironment and ultimately influence clinical outcomes.

LDHA, a key terminal enzyme in glycolysis that catalyzes the conversion of pyruvate to lactate, is a major contributor to the lactate-enriched TME. Accumulating evidence indicates that excess lactate promotes immunosuppression through multiple non-mutually exclusive mechanisms, including microenvironmental acidification, metabolic competition, activation of HIF-1α-related pathways, and the recently described epigenetic mechanism of protein lactylation, which translates metabolic cues into transcriptional reprogramming of macrophages [6,7]. These intertwined metabolic and epigenetic processes bias TAMs toward an M2-like phenotype and support pro-tumorigenic programs. Accordingly, incorporating LDHA into our prognostic model is both statistically justified and biologically plausible, as it represents a central metabolic node within the immunosuppressive TME. LDHA may represent a central metabolic node underlying the “high-lactate, immunosuppressive” TME in HCC, capable of coordinating TAM functional states through intertwined metabolic, epigenetic, and intercellular communication mechanisms, thereby potentially influencing immunotherapy sensitivity and clinical outcomes.

HNRNPU is a nuclear RNA-binding protein and nuclear-matrix-associated factor whose role in HCC has been increasingly recognized in recent years. Accumulating evidence highlights the potential clinical relevance of HNRNPU in HCC. HNRNPU is upregulated in HCC, and its knockdown suppresses tumor cell proliferation and disrupts transcriptional programs associated with malignant progression [8]. These findings suggest that HNRNPU may serve not only as a biomarker but also as an active regulator of tumor phenotypes. Building on our observed associations with immune infiltration features, a plausible inference is that *HNRNPU*—through transcriptional and/or splicing regulation—may help shape gene expression programs related to immune cell recruitment, inflammatory cytokine networks, or metabolic adaptation, thereby potentially coupling tumor-intrinsic regulatory states to the immune contexture of the TME. This hypothesis, however, requires direct experimental validation, particularly with respect to its effects on chemokine axes, TAM lineage specification, and metabolism-related gene networks.

Taken together, the prognostic model genes can be interpreted as capturing three complementary dimensions of immunometabolic dysregulation in HCC: LDHA reflects lactate-driven metabolic stress and immunosuppressive signaling; NPM1 represents tumor-intrinsic alterations in antigen presentation and immune-evasion circuitry; and *HNRNPU* may encode adaptive programs at the level of transcriptional and/or splicing regulation. This integrated view strengthens biological plausibility of the prognostic model and provides a clearer framework for downstream mechanistic interrogation. In the nomogram, pathological stage and the risk score contributed the majority of prognostic points, indicating their dominant impact on survival prediction, whereas age and gender showed comparatively smaller effects. From a translational perspective, the prognostic model developed in this study may facilitate more refined risk stratification and outcome prediction and could help identify patient subgroups less likely to benefit from immunotherapy. In addition, therapeutic strategies targeting the lactate axis and its associated regulators (e.g., LDHA, HNRNPU, and NPM1) may represent a potential avenue for modulating the immune microenvironment and improving treatment responsiveness, although these possibilities require further mechanistic and clinical validation.

Paeoniflorin has demonstrated immunomodulatory potential in experimental HCC models, including modulation of PD-L1 expression and T cell–related responses [10]. However, whether it directly engages lactate metabolism remains unclear. Our docking and MD analyses suggest structural compatibility between paeoniflorin and LDHA, but these findings indicate only a potential interaction rather than confirmed biochemical inhibition. We therefore frame LDHA as a candidate target that warrants further biochemical and in vivo validation.

Building on the above integrative analyses, we propose the following testable central hypothesis. Paeoniflorin may potentially interact with LDHA at the structural level, raising the possibility that it could influence lactate-associated pathways, which warrants further biochemical and cellular validation. Concurrently, dampening the lactate axis may reshape *HNRNPU/NPM1*-linked transcriptional and immune-evasion programs—particularly those governing antigen-presentation suppression—potentially contributing to modulation of the HCC immune microenvironment, pending experimental validation. Conceptually, our study reinforces the emerging framework that lactate metabolism is a driver of immunosuppression in HCC and further suggests that jointly considering the lactate axis (LDHA and downstream lactylation/transport processes) together with tumor-intrinsic immune-evasion mechanisms (e.g., NPM1-mediated alterations in antigen presentation) may more faithfully capture the multilayered immunosuppressive architecture of the TME, thereby offering a new perspective for understanding heterogeneity in immunotherapy responsiveness [9].

Despite our efforts to integrate multi-dimensional data, several limitations warrant consideration. Public datasets may be confounded by batch effects, platform heterogeneity, and variability in sample processing. Moreover, immune deconvolution methods such as ESTIMATE and ssGSEA depend on predefined gene signatures and computational assumptions, which can introduce systematic uncertainty and limited resolution in estimating specific immune cell populations. Accordingly, these results should be interpreted as trend-based inferences rather than precise quantifications of immune infiltration. Future studies incorporating multiple deconvolution algorithms may further improve analytical robustness and reliability. In addition, although docking and MD analyses suggest structural feasibility, their functional effects must be further confirmed through biochemical assays, metabolic flux analyses, and cell-based experimental validation. Importantly, the prognostic model was developed and internally validated exclusively within the TCGA-LIHC cohort, and no independent external validation cohort (e.g., ICGC or GEO datasets) was included in the present study. Therefore, the generalizability and robustness of the model gene signature across different populations and sequencing platforms remain to be established. The absence of external validation introduces a potential risk of overfitting, and the predictive performance observed in this discovery cohort should be interpreted cautiously. Future studies incorporating multi-center cohorts and prospective validation are warranted to confirm the clinical utility and stability of this model. Overall, from the perspective of natural-product target discovery, our structural results link paeoniflorin to LDHA as a prioritized candidate target and outline an actionable framework for subsequent pharmacological investigations that bridge natural compounds, metabolic enzymes, and the immune microenvironment. Collectively, we propose a lactate-centered immunometabolic regulatory axis potentially targetable by paeoniflorin in HCC.

## 4. Materials and Methods

### 4.1. Data Acquisition and Preprocessing

Transcriptomic RNA-seq data (raw counts) and corresponding clinical information for HCC were downloaded from TCGAdatabase using the R package TCGAbiolinks (version 2.30.0) [11]. After excluding samples with missing clinical data, 368 tumor samples and 50 adjacent normal samples were retained for subsequent analyses. To improve comparability across samples, raw counts were transformed into FPKM values using DESeq2 (version 1.42.0). Detailed clinical annotations (Table 2) matched to the samples were obtained from the UCSC Xena platform (accessed on 20 November 2025) [12]. Potential PF targets were compiled by integrating multiple databases. Briefly, “Paeoniflorin” was queried in the HERB database (accessed on 20 November 2025) [13] and SymMap (accessed on 20 November 2025), yielding 77 and 48 targets, respectively. After merging and removing duplicates, 107 PF candidate targets were obtained (Table S1). HCC-related disease targets were retrieved from the Comparative Toxicogenomics Database (CTD; accessed on 20 November 2025) using “Liver Cancer” as the query term. Targets with an interaction count > 40 were retained, resulting in 9392 high-confidence HCC-related targets (Table S2). A macrophage- and lactylation-related gene set was established as follows:

MRGs and LRGs were identified by searching GeneCards [14] using relevant keywords. Using “Macrophage” as the keyword, 4169 protein-coding genes [15] with a relevance score > 1 were retrieved, and 25 additional MRGs were curated from PubMed-indexed literature, yielding a total of 4187 MRGs after removing duplicates. For lactylation-related genes, 113 LRGs were collected using “Lactylation” as the keyword in GeneCards, with an additional 332 genes compiled from published literature [16,17,18,19,20,21]. After merging and removing duplicates, 427 LRGs were retained. Finally, the intersection of MRGs and LRGs was defined as MLRGs, comprising 221 genes (Table S3).

The integration of GeneCards retrieval with literature curation was adopted in consideration of the current lack of a standardized gene set for lactylation-related genes. We also reviewed the MSigDB and Gene Ontology (GO) databases; however, due to the absence of a dedicated gene set specifically annotated for lactylation-related functions, these resources were not directly utilized in the present study.

### 4.2. Identification of Macrophage- and Lactylation-Related Differentially Expressed Genes

According to the sample grouping of the TCGA-LIHC dataset, samples were divided into the liver cancer (LIHC) group and the control (Control) group. The R package DESeq2 [22] (version 1.42.0) was used to perform differential gene expression analysis between the LIHC group and the control group. A DESeqDataSet object was constructed using the DESeqDataSetFromMatrix function. Normalization and differential expression analysis were performed with the DESeq function. *p* values were adjusted for multiple testing using the Benjamini–Hochberg (BH) method, as implemented by default in DESeq2. Genes with |logFC| > 0 and adjusted *p* < 0.05 were considered upregulated, whereas genes with logFC < 0 and adjusted *p* < 0.05 were considered downregulated. The *p*-value correction method was the Benjamini–Hochberg (BH) method. The results of the differential analysis were visualized using the R package ggplot2 (version 3.4.4) to generate volcano plots. To obtain MLRDEGs associated with LIHC and paeoniflorin, all DEGs with |logFC| > 0 and adjusted *p* < 0.05 identified in the TCGA-LIHC dataset were intersected with paeoniflorin targets, LIHC disease targets, and MLRGs, and the overlap was visualized using a Venn diagram. The resulting MLRDEGs were identified, and a heatmap was generated using the R package pheatmap (version 1.0.12). The analysis scripts are available upon reasonable request to enhance the transparency and reproducibility of the study.

### 4.3. Gene Ontology and Kyoto Encyclopedia of Genes and Genomes Enrichment Analyses

Functional annotation and pathway enrichment analyses for MLRDEGs were performed using the R package clusterProfiler (version 4.10.0) [23]. Gene Ontology (GO) analyses encompassed the BP, CC, and MF categories [24], and KEGG pathway enrichment analysis was also conducted [25]. Enriched terms were considered statistically significant if adjusted *p* < 0.05 and FDR (*q* value) < 0.25, with BH correction applied.

### 4.4. Protein–Protein Interaction Network

MLRDEGs were submitted to the STRING database (version 11.5; accessed on 20 November 2025) [26] to construct a PPI network using a medium-confidence threshold (minimum interaction score > 0.400). The network was visualized and analyzed using Cytoscape (version 3.10.0) [27]. GeneMANIA (accessed on 20 November 2025) [28] was used to predict functionally similar genes and to construct a supplementary functional association network.

### 4.5. Construction of the Prognostic Risk Model and Survival Analyses

LASSO regression analysis was performed using the R package glmnet [29], (Version 4.1-8) with the parameter family = “cox”, based on MLRDEGs identified in the univariate Cox regression analysis. The core function cv.glmnet was applied with 10-fold cross-validation (nfolds = 10) to determine the optimal regularization parameter (λ). The final model was selected according to lambda.min, corresponding to the λ value that yielded the minimum cross-validation error. To reduce the influence of random variation, the procedure was repeated 10 times (iter.times = 10), and the gene combination with the highest selection frequency was defined as the final model. The corresponding regression coefficients were subsequently extracted. LASSO regression extends linear regression by incorporating a penalty term (λ × the absolute value of the coefficients), thereby reducing overfitting and improving model generalizability.

The results of the LASSO analysis were visualized using the prognostic risk model plot and the coefficient trajectory plot. Finally, multivariable Cox regression analysis was conducted based on the LASSO-derived risk score and clinical variables. The risk score was calculated as follows:

$$risksore=\sum_{i}Coefficient\left({gene}_{i}\right)*mRNA\;Expression\;({gene}_{i})$$

Patients were stratified into high- and low-risk groups based on the median risk score. Kaplan–Meier survival curves were generated using the survival package (version 3.5-7) [30,31], and group differences were evaluated using the log-rank test. Time-dependent ROC curves were generated using the R package timeROC (version 0.4) to calculate AUCs for 1-, 2-, and 3-year survival, thereby assessing predictive performance [32]. The analysis scripts are available upon reasonable request to enhance the transparency and reproducibility of the study.

### 4.6. Differential Expression Validation and ROC Analyses of Prognostic Model Genes

Expression differences in the prognostic model genes between HCC and normal samples were evaluated using the Wilcoxon rank-sum test. ROC curves for individual prognostic model genes were generated using the R package pROC (version 1.18.5) [33], and AUC values were calculated to assess their diagnostic performance.

### 4.7. Tumor Immune Microenvironment and Immune Infiltration Analyses

The IOBR package (version 0.99.9) [34] was used to calculate immune scores, stromal scores, and ESTIMATE scores for each sample based on the ESTIMATE algorithm. Differences between HCC and normal controls were compared.

ssGSEA was employed to quantify the relative enrichment scores of immune cell subpopulations. Each infiltrating immune cell type was annotated (e.g., activated CD8 T cells, activated dendritic cells, gamma delta T cells, natural killer cells, and regulatory T cells). Single-Sample Gene Set Enrichment Analysis (ssGSEA) was performed using the R package GSVA [35] (version 1.48.0). Parameters were specified via the ssgseaParam function, with default settings including kcdf = “Gaussian” and mx.diff = TRUE. The gsva() function was subsequently applied to calculate enrichment scores, generating an immune cell infiltration matrix that represents the enrichment score of each immune cell type for each sample. Group comparisons were visualized using the R package ggplot2 (version 3.4.4) to illustrate differences in immune cell infiltration between the LIHC group and the control group in the TCGA-LIHC cohort. Immune cell types with significant differences between groups were retained for subsequent analysis, and correlations among immune cells were calculated using Spearman’s correlation analysis.The R package pheatmap (version 1.0.12) was used to generate a correlation heatmap illustrating the relationships among immune cell types. Correlations between the prognostic model genes and immune cell types were calculated using Spearman’s correlation analysis, and results with *p* < 0.05 were retained. The R package ggplot2 (version 3.4.4) was used to generate correlation bubble plots illustrating the associations between the prognostic model genes and immune cell types [36]. The analysis scripts are available upon reasonable request to enhance the transparency and reproducibility of the study.

### 4.8. Molecular Docking

The three-dimensional (3D) structure of PF (CID: 442534) was downloaded from PubChem [37]. Crystal structures of LDHA (PDB ID: 4JNK) and NPM1 (PDB ID: 7OBG) were obtained from the RCSB PDB database (accessed on 20 November 2024) [38]. For HNRNPU, the high-confidence predicted structure (pLDDT > 70) was downloaded from the AlphaFold Protein Structure Database (accessed on 20 November 2024) [39].

Semi-flexible docking was performed using the CB-Dock2 website (accessed on 20 November 2025) [40], which integrates AutoDock Vina [41,42](AlphaFold Protein Structure Database). CB-Dock2 is an improved version of CB-Dock server for blind docking of protein ligands, which integrates cavity detection, docking and homology template fitting into one. Docking affinity was reported as the Vina score (kcal/mol), where lower values indicate more stable binding; a Vina score < −7.0 kcal/mol was considered suggestive of strong binding. Representative poses were visualized using PyMOL (version 2.5.0).

### 4.9. Molecular Dynamics Simulation

The complex with the best docking affinity (lowest Vina score) was selected for MD simulation [43]. Simulations were performed using GROMACS (version 2022). The system was solvated using the TIP3P water model, and ions were added to neutralize the system. Simulations were conducted under constant temperature (310 K) and constant pressure (101 kPa) with periodic boundary conditions. After energy minimization, equilibration was conducted under NVT and NPT ensembles (100 ps each), followed by a 50 ns production run at 310 K and 1 bar; trajectories were saved every 10 ps. Trajectories were analyzed using GROMACS built-in tools and custom scripts, including root mean square deviation (RMSD; backbone stability), root mean square fluctuation (RMSF; residue flexibility), radius of gyration (Rg; compactness), and solvent-accessible surface area (SASA).

### 4.10. Statistical Analysis

All statistical analyses were performed using R (version 4.3.3). For comparisons of continuous variables between two groups, Student’s *t*-test was used when the data were normally distributed; otherwise, the Mann–Whitney U test (Wilcoxon rank-sum test) was applied. Comparisons among three or more groups were conducted using the Kruskal–Wallis test. Spearman’s rank correlation coefficient was calculated for correlation analyses. Survival differences were assessed using the log-rank test. A two-sided *p* < 0.05 was considered statistically significant.

## Figures and Tables

**Table 1:** Results of GO and KEGG enrichment analysis for MLRDEGs.

Ontology	ID	Description	Gene Ratio	Bg Ratio	*p* Value	p. Adjust	q Value
BP	GO:0032886	regulation of microtubule-based process	4/8	249/18,800	2.02 × 10^−6^	0.001864	0.000679
BP	GO:0034644	cellular response to UV	3/8	88/18,800	5.46 × 10^−6^	0.002192	0.000799
BP	GO:0071482	cellular response to light stimulus	3/8	121/18,800	1.42 × 10^−5^	0.003286	0.001198
BP	GO:0009411	response to UV	3/8	146/18,800	2.5 × 10^−5^	0.003742	0.001364
BP	GO:0010970	transport along microtubule	3/8	157/18,800	3.1 × 10^−5^	0.003742	0.001364
CC	GO:0032839	dendrite cytoplasm	2/8	30/19,594	6.31 × 10^−5^	0.004543	0.002723
CC	GO:0120111	neuron projection cytoplasm	2/8	89/19,594	0.000561	0.020202	0.012109
CC	GO:0000922	spindle pole	2/8	169/19,594	0.002001	0.045622	0.027347
CC	GO:0032838	plasma membrane bounded cell projection cytoplasm	2/8	220/19,594	0.003361	0.045622	0.027347
CC	GO:0099568	cytoplasmic region	2/8	259/19,594	0.004624	0.045622	0.027347
MF	GO:0003712	transcription coregulator activity	3/8	497/18,410	0.00099	0.014021	0.004167
MF	GO:0005507	copper ion binding	2/8	61/18,410	0.000299	0.009903	0.002943
MF	GO:1990841	promoter-specific chromatin binding	2/8	62/18,410	0.000308	0.009903	0.002943
MF	GO:0002039	p53 binding	2/8	66/18,410	0.00035	0.009903	0.002943
MF	GO:0016209	antioxidant activity	2/8	85/18,410	0.000579	0.012311	0.003659
KEGG	hsa04213	Longevity-regulating pathway—multiple species	2/7	62/8164	0.001163	0.031559	0.023576
KEGG	hsa04211	Longevity-regulating pathway	2/7	89/8164	0.002382	0.031559	0.023576
KEGG	hsa04922	Glucagon signaling pathway	2/7	107/8164	0.003424	0.031559	0.023576
KEGG	hsa04152	AMPK signaling pathway	2/7	121/8164	0.004358	0.031559	0.023576
KEGG	hsa04068	FoxO signaling pathway	2/7	131/8164	0.00509	0.031559	0.023576

GO, Gene Ontology; BP, Biological Process; CC, Cellular Component; MF, Molecular Function; KEGG, Kyoto Encyclopedia of Genes and Genomes; MLRDEGs, macrophage- and lactylation-related differentially expressed genes.

**Table 2:** Overall Baseline Data Sheet.

Characteristics	Overall
OS, *n* (%)	
1	164 (39.2%)
0	254 (60.8%)
OS.time, median (IQR)	608 (347, 1148.5)
DSS, n (%)	
1	97 (23.8%)
0	310 (76.2%)
DSS.time, median (IQR)	608 (347, 1148.5)
Age, *n* (%)	
≥65	181 (43.3%)
<65	237 (56.7%)
Gender, *n* (%)	
MALE	277 (66.3%)
FEMALE	141 (33.7%)
Stage_Pathologic, *n* (%)	
Stage I	190 (49.2%)
Stage II	96 (24.9%)
Stage III&IV	100 (25.9%)

## Data Availability

The RNA-seq transcriptomic data and corresponding clinical information analyzed in this study were obtained from The Cancer Genome Atlas (TCGA) Liver Hepatocellular Carcinoma cohort (TCGA-LIHC) and are publicly available via the Genomic Data Commons (GDC, https://gdc.cancer.gov/) (accessed on 20 November 2025). Clinical annotations matched to samples were also accessed through the UCSC Xena platform (accessed on 20 November 2025). Paeoniflorin-related targets were collected from HERB and SymMap databases (accessed on 20 November 2025), HCC disease targets were retrieved from the Comparative Toxicogenomics Database (CTD; accessed on 20 November 2025), and macrophage-/lactylation-related gene sets were compiled from GeneCards and published literature (as described in the Methods). The 3D structure of paeoniflorin was obtained from PubChem, and protein structures were obtained from the RCSB PDB and the AlphaFold Protein Structure Database. All data supporting the findings of this study are included in the article and/or provided in the Supplementary Tables (e.g., Tables S1–S4). The analysis scripts supporting the findings of this study are included in the Supplementary Materials.

## Supplementary Materials

The following supporting information can be downloaded at: https://www.mdpi.com/article/10.3390/ijms27052495/s1.

## Abbreviations

TCGA	The Cancer Genome Atlas
LIHC	Liver Hepatocellular Carcinoma
DEGs	Differentially Expressed Genes
MLRGs	macrophage- and lactylation-related genes
MLRDEGs	macrophage- and lactylation-related differentially expressed genes
CTD	Comparative Toxicogenomics Database
GO	Gene Ontology
KEGG	Kyoto Encyclopedia of Genes and Genomes
LASSO	Least Absolute Shrinkage and Selection Operator
ROC	Receiver Operating Characteristic
ESTIMATE	Estimation of Stromal and Immune cells in Malignant Tumor tissues using Expression
ssGSEA	Single-Sample Gene Set Enrichment Analysis
BP	biological process
CC	cellular component
MF	molecular function
PPI	network, Protein–protein interaction Network
OS	Overall Survival; KM, Kaplan–Meier
AUC	Area Under the Curve
TPR	True Positive Rate
FPR	False Positive Rate
