# Supplementary material for: Bioinformatics Profiling of a Paeoniflorin-Associated “Macrophage–Lactylation” Axis in Hepatocellular Carcinoma: An LDHA-Based Prognostic Model and Structural Computational Evidence
Source: Int J Mol Sci. 2026 Mar 9;27(5):2495. doi: 10.3390/ijms27052495 (PMC12986180; doi:10.3390/ijms27052495)
Supplement: Supplementary file 1 [file ijms-27-02495-s001.zip › Supplementary File/ijms-4165836-Table Supplementary1-4.zip!/ijms-4165836-Table Supplementary1-4/ijms-4165836-Supplementary Table1.pdf]

gene
ALPP
IL10
NLRP3
PPARGC1A
IL1B
MET
PRKAA2
NFKB1
GSDMD
CASP3
SOD2
HDAC4
JUN
FOXO1
GDNF
TLR4
SOCS3
IL37
AXL
CYCS
CASP9
IFNG
NR1H4
FBXO32
FAS
C1QA
PRKAA1
PRKAB1
IL12RB1
STK11
ICAM1
BCL2L1
EPX
HSPA4
SOD1
LTB4R
HSPA8
MAPK14
PMPCA
MAP3K5
MEF2A
IL1A
AKT1
SUMO1
IGHE
PLCG1
COL1A1
ACTA2
IL18
MMP13

CRP
SIRT1
BDNF
SREBF1
MYOG
HNRNPU
NPM1
CAT
SMAD2
MYOD1
KDR
IL6
TNFRSF1A
KMT2B
MAPK8
IL4
PIK3CA
SLC16A1
PPARG
TNF
CASP1
F3
HSPA5
TGFB1
CASP7
GAS6
LDLR
ADA
ALB
GFAP
IGF1
INS
JAK1
JAK2
JAK3
LDHA
MAPK1
MAPK3
MPO
MVK
NGF
PMVK
STAT1
STAT3
TCF3
TYR
TYRP1
CD14
LBP
PRL
SOD3

PPO2
ADORA1
CNR2
GPX2
GPX3
HTR1D
