# Supplementary material for: Bioinformatics Profiling of a Paeoniflorin-Associated “Macrophage–Lactylation” Axis in Hepatocellular Carcinoma: An LDHA-Based Prognostic Model and Structural Computational Evidence
Source: Int J Mol Sci. 2026 Mar 9;27(5):2495. doi: 10.3390/ijms27052495 (PMC12986180; doi:10.3390/ijms27052495)
Supplement: Supplementary file 1 [file ijms-27-02495-s001.zip › Supplementary File/ijms-4165836-Table Supplementary1-4.zip!/ijms-4165836-Table Supplementary1-4/ijms-4165836-Supplementary Table2.pdf]

gene
CCND1
PARP1
CASP8
JUN
MKI67
CAT
NFE2L2
MYC
EGFR
CYP1A1
AFP
CCNB1
TNF
TGFB1
CYP1A2
SOD2
AKT1
MMP2
CTNNB1
IL6
IGF1
EGF
CDK1
GSTM1
PTGS2
SREBF1
RB1
FOS
HSPB1
PCK1
SOD1
PTEN
EGR1
CEBPB
ESR1
VCAM1
CDKN1B
CCNE1
CYP2E1
MMP9
CXCL12
GDF15
CDK4
EPHX1
TRP53
HMGCR
TOP2A
GSTP1
FASN
HSPA5

CEBPA
ACACA
STAT1
ABCB1
CTSD
HGF
DPYD
BID
SLC2A2
RRM2
MTOR
SLC2A1
NR1H4
TLR4
MET
CDC20
CP
E2F1
IRS1
DCN
TGFA
HRAS
IGF1R
ACOX1
UBE2C
CENPE
BTG2
CCN1
CCNB2
SCD
CEBPD
MPO
AURKA
IGF2
NR0B2
MAD2L1
ABCB4
HMGB2
TK1
RPS6
ME1
PDK4
CRP
ANXA4
SOCS3
LCAT
PDIA3
CYP17A1
TYMS
CDC25C
TERT

GHR
CBR1
MCM2
APOA1
CCL3
CDKN3
GLI1
RRM1
ISG15
NUSAP1
HSPA9
RARA
PKM
KRAS
GLUL
PBK
CDKN2B
MT1A
UBD
USP2
KIF2C
CDKN2A
IRS2
MT2A
KIF20A
PTTG1
CENPF
PLK1
PHLDA1
CCNF
SNAI1
GJB1
TH
SFN
SOCS2
AKR1B10
ACTB
GAPDH
ATG7
CDKN2C
STMN1
ENO1
CDC6
BUB1
A2M
CXCL14
SREBF2
FDFT1
GNMT
UHRF1
NME1

CENPA
IL1RN
PDGFB
EZH2
F2
PRC1
TRIM24
EGR2
ACSL4
MAT1A
ZWINT
APEX1
FOXM1
TTK
RAC1
ACLY
FST
HAO2
PGD
PTK2
LIFR
ATM
DLGAP5
NDC80
AURKB
ASPM
PRDX2
IGFALS
SLC22A1
APC
EPAS1
BUB1B
MVK
CD34
TGFB2
ECT2
ACO2
FABP5
PRDX1
FBP1
NUF2
PYCARD
TALDO1
MMP14
DTL
ECM1
TPX2
IL1RAP
ASF1B
PZP
CDH13

SKP2
RACGAP1
RCAN1
KIF11
CDCA3
IDH1
KIF23
PIK3CA
PYGL
NCAPH
ESM1
CYP39A1
NEK2
SPC25
BSG
MCM10
APCS
TFPI2
MAPT
CDCA8
ZFP36
E2F8
COMT
FOSB
ADH4
TRIP13
ANLN
CD163
INMT
CDCA2
CDCA5
ROBO1
EREG
HMMR
CENPU
ETFA
ERP29
CHAF1B
PRDX6
TCF19
CFP
CSPG4
SRPX
CA2
HAMP
UCHL1
GMNN
PLAC8
RND3
NCAPG
STAT4

CD5L
LRAT
NNMT
ADAMTS1
PDGFRL
CEP55
CYP2B6
YTHDF2
THY1
IGF2R
PGK1
KBTBD11
IRF2
FANCI
MELK
NR1H2
UMPS
TST
COL15A1
TAGLN2
AADAT
CCNA1
VASN
OLFML2B
MYBL2
SFRP1
KIF15
GTSE1
KIF4A
RPS6KA3
AKR1C2
CDC45
AKR1A1
MECP2
KMT2A
IGF2BP3
EHD3
IQGAP1
DEPDC1B
SERPINA1
SHH
FGFR1
TACC3
IQGAP2
PCLAF
UBE2T
MED1
C9
RAD54L
BRAF
HHIP

KIF14
BMPER
DEPDC1
GPC3
LYVE1
KMT2C
ARHGAP11A
NAT2
YAP1
APLN
BCO2
KIFC1
CYLD
CCR1
MLXIPL
TROAP
PKMYT1
MALAT1
PTH1R
KCNN2
HSD3B2
PAMR1
NOTCH3
ITIH3
MARCO
MBTPS1
CENPM
ARID1B
CDK14
EAF2
CELSR3
SLC25A47
CDT1
TSC1
CSRNP1
FANCD2
EXO1
IQGAP3
TCIM
CD274
GYS2
ASPG
SIK2
OLFML2A
PNPT1
HGFAC
TSLP
INPP4B
PHB1
RUNX3
ARID2

KIF18A
FAM83D
SCAP
CNDP1
CENPW
SLIT2
PPP1R1A
SKA1
RSPO3
CAP2
CYP2C8
CKAP2L
ATP5PD
TLR9
AXIN1
GINS1
PITX1
COPS5
ANGPTL6
ARID1A
UROC1
PARK7
DNAJC6
VIPR1
GNAO1
JDP2
UBE2E2
CPEB3
MT1E
ND5
MFSD2A
APOF
ADD1
CCBE1
PLVAP
FGF4
TLR7
RTP3
USP4
GPM6A
FGF19
TRAIP
PKP1
NEIL3
LRRC1
CEP131
MBTPS2
COLEC10
HERC5
KMT2B
ADRA2B

ORC1
FAM111B
WDR62
KDM8
STAB2
VSIG4
MRO
ZIC2
MICA
TP53
BIRC5
BCL2L1
CXCL8
PPARG
TNFSF10
AR
NFKBIA
HTATIP2
ACE
SERPINA4
SLC5A5
CD276
IFNA1
CDKN1A
PCNA
CASP3
FAS
BAX
NFKB1
GADD45A
NQO1
RELA
VEGFA
ABCC3
IL1B
CASP9
GPT
GSR
CD36
MDM2
BCL2
CD14
MAPK1
CDH1
COL1A1
TRIB3
STAT3
H2AX
GPX1
ABCC2
GSK3B

IFNG
ALDH1A1
GSTA2
DDIT3
HMOX1
MAPK3
KRT8
MAPK8
PPARA
CYCS
CDH2
VIM
GCLC
TIMP1
MAPK14
DNMT1
ACTA2
IL10
ICAM1
CCN2
CCNA2
CASP7
MGMT
COL1A2
CCNG1
GSTM3
ABCG2
GADD45B
ATF3
UGT1A1
NR3C1
ODC1
IGFBP3
CDK2
SCD1
GOT1
NR1H3
HPRT1
HIF1A
SPP1
AHR
S100A9
CYP3A11
HES1
GSTA4
POR
TNFRSF10B
FGF2
APAF1
NOS2
XIAP

IL18
CXCL10
NR1I2
INSIG1
FN1
CXCR4
ID1
FASLG
ERBB2
LDHA
PPARGC1A
ABCA1
ALB
ALPL
CYP2B10
SLC3A2
BECN1
GPAM
TFRC
SQLE
BIRC3
CCND2
TIMP2
CPT1B
BAD
CXCL1
SRXN1
GSTA1
CPT1A
IGFBP1
IL1A
LEP
ABCC1
APP
XBP1
SRC
GJA1
SERPINE1
XDH
ABCC4
SPARC
F3
MAPK9
EHHADH
GCLM
BAK1
GSTM2
TPM1
HSP90AB1
HSPD1
PDGFRB

LHB
SGK1
DDIT4
RPS6KB1
FOSL1
EPCAM
TGFB3
DUSP1
PKLR
CCL2
S100A8
GPX2
EFNA1
MAP1LC3B
IER3
CYP7A1
SLCO2B1
ID3
ABCB1A
RAD51
TGFBR2
CCL5
ALAS1
HSPA1A
MAP2K1
GSTT1
LPL
ASNS
CYP1B1
CCND3
PLAU
NDRG1
SQSTM1
CASP4
ARG1
NRG1
ENPP2
VLDLR
ACSL1
MMP3
EIF4EBP1
SP1
IGFBP4
NR1I3
CFLAR
HMGCS1
FSTL1
HSPE1
COL3A1
BDNF
CAV1

UBC
IGFBP2
KEAP1
GAS6
TXNIP
ECH1
FABP4
CTH
BCL6
LGALS3
PSAT1
XRCC1
SMAD3
PRKCB
ID2
DECR1
TJP1
CASP1
LUM
PIM1
KDR
AK4
MAT2A
CYP4A10
DNMT3A
ANGPTL4
CTSB
CCNG2
IHH
NFIL3
ESR2
ACADL
CYP7B1
SESN2
TLR2
RUNX2
DNMT3B
SLC7A11
SULT1A1
TNFRSF1A
ENC1
PTGS1
S100A4
STAR
CYP4A14
ATF4
WEE1
CDKN1C
TSC22D1
UGT1A6
CXCL2

C1S
CLU
IL4
ALAD
HSPH1
OAT
SAT1
EDN1
IRF7
RARB
XRCC5
ANXA3
PTGES
CYP2B1
RHOB
ADD3
PROM1
BNIP3
G6PC1
KRT18
CASP12
RXRA
IGFBP5
PPP2CA
NUPR1
NPM1
TGFBI
PTP4A1
TXNRD1
ASS1
PDGFA
PLK2
FSHB
CYP3A23-3A1
FKBP5
PLAT
PHGDH
ACADM
MCL1
SMOX
PLSCR1
LPIN1
BBC3
DGAT2
PER2
DLC1
CYP3A4
SLCO1A1
CALR
APOE
ABCG5

RGS2
ANXA5
LITAF
SDHA
BHLHE40
DUSP6
FMO5
MCM6
MMP7
MFGE8
CLDN1
MT1
SLCO2A1
JAK1
NCL
PMAIP1
PRKCA
CTSL
CHEK1
PPP1R15A
THBS1
THBD
GSTA3
ADM
ITGB1
HNF4A
ABCC5
SCARB1
CFB
RBP1
GGT1
PRKAA1
KRT19
SOX9
PGR
PDK1
KIT
GRB10
NAT8
CREB1
IL2
BDH1
BMP2
GADD45G
AQP7
PTCH1
PECAM1
FABP1
NRP1
ABCB1B
AIFM1

PLIN2
PON1
NT5E
ATP5F1B
NEDD9
CAR3
SDC2
HMGCS2
HAL
IFITM1
G0S2
CSF2
PPARD
CST3
CTSC
HSD3B1
CASP6
MSH2
CD44
SULF2
TNFRSF21
ACOT1
NOX4
ISYNA1
CREBBP
CTSS
BRCA1
DNM1L
MYD88
SERPINE2
LEPR
SELENOP
FADS2
SRD5A1
TAP1
SLC40A1
GPX4
ITGAV
GRN
TAGLN
TGFBR3
OCLN
ZEB2
CYBA
NOS3
G6PD
IL1R1
IKBKB
COL6A3
MAP2K6
MSMO1

CROT
HSPA2
KLF5
EIF5
AXIN2
BIK
TUBB2A
GPX3
CITED2
LDLR
DST
LY6E
FBXO32
AKR1B1
CYP11A1
FOXA1
NAP1L1
EEF1A1
EIF2AK3
JAG1
ABCB11
CKB
SMOC1
CYP26A1
JUNB
SNCA
PMP22
CD68
GSN
ALDH2
AREG
FTH1
PON2
PLA2G4A
EDNRB
AXL
ACACB
PLTP
KRT23
IL12B
ERN1
BCL2L11
RGS16
CHUK
NOTCH1
AGT
SLC10A1
EIF2S1
GULO
CHAC1
HERPUD1

RARRES1
ZFP36L1
SLC22A5
CAPN2
NTRK2
ENTPD5
FABP3
PHLDA2
PLA2G2A
POSTN
HSP90B1
ITGAM
RAF1
UGDH
F2R
KLF6
PAICS
CLDN4
TRIB1
INS
BIRC2
PDLIM1
NFKB2
PDGFRA
ACO1
SMC2
STAT5B
LOX
LXN
MAP2K3
PLK3
SPON2
MGLL
SLC16A1
SELE
APOA4
PRKCZ
GBP2
OGG1
NLRP3
FUBP1
FHL2
PPP1R3C
GSS
MMP1
ATF5
JAK2
ACOT2
MCM7
MAP2K2
SIRT1

FADS1
CD74
RBBP4
DNAJB1
HSPA8
ANXA2
COL4A1
ETS2
ADIPOQ
CDK6
PNRC1
KLF4
HMGB1
HSDL2
VNN1
BGLAP
CPT2
CSF1
DIO2
KNG1
UCP2
DBI
ERCC1
IL17A
TMPO
ATP2A2
TYROBP
TF
HSP90AA1
PNPLA2
GFAP
SLC2A4
SULT2A1
CD9
ACAA2
CD38
DDAH1
ACSS2
COL6A1
PRKCD
INS1
ACAT1
TNC
DNAJB4
STC2
AHCY
MAP3K6
GDF10
PIR
MASP1
ADA

FBLN1
MAFF
S100A6
COL18A1
NCF1
PRMT1
HSPA4L
TUBA1C
FGF7
POLG2
JUND
SLC20A1
HDAC1
RAMP1
PDIA4
S100A11
THRSP
APOB
PCTP
ALCAM
CYP27A1
TXNL1
BHMT
HSPA1B
PLCB1
ACSL5
PHLDA3
FGF1
MCM5
ADH1
POLA1
MBP
NID1
HSPB8
SDC4
SMAD2
CYP19A1
GLO1
DKK3
ETS1
HSPA4
ALDH1A7
GSTA5
PFKP
RPSA
CYP3A13
FOXO1
ANXA1
HSD17B4
MCM4
GSTM5

RCAN2
CYBB
RBP4
FAT1
BMF
GPM6B
FDPS
SLC25A30
BTG1
GSTO1
ECI1
PAPSS2
SLC25A4
PLK4
GUSB
POU5F1
PDCD4
PRLR
PIGR
ALOX5
CTSH
MME
CKS1B
RAD51AP1
TNFRSF12A
TPM3
MYBL1
VDR
C3
BGN
CSF1R
BCL3
DAPK1
IL4R
CYB5A
DDC
TGM2
NFIA
ALDH7A1
MXI1
TNFRSF1B
ATP8B1
B2M
SOX4
SEPHS2
ELOVL6
GLB1
SERPINB2
ATP6V1A
SFRP2
TIMELESS

SLC7A5
INHBE
P4HB
ADGRE1
LGALS1
CYP26B1
MARCKS
INHBA
CLDN2
INSIG2
LGMN
TP53INP1
AQP3
TSC22D3
PLAUR
CDC34
DAB2
RORA
RSAD2
DSC2
MGP
NREP
PPL
SOD3
CHKA
TYMP
KYNU
S1PR1
BMAL1
CKS2
STIP1
TNFAIP3
ARRDC2
DCXR
DIABLO
RTN4
HSF1
PALLD
MAP3K5
CYP2C39
SLPI
PALMD
SH3BGRL3
OSGIN1
SERPINH1
GPNMB
NR1D1
ABCG1
ABCC6
PEX11A
VAT1

CPEB2
COTL1
SLC39A8
CKM
ANXA6
CRAT
SMAD7
UNG
CBS
MVP
TMBIM1
DHCR24
MT2
DUSP10
FUS
PNPLA3
ADAM17
SLC27A2
CSRP3
GATA3
NANOG
TNFSF11
S100A10
CD40
HPGD
LEF1
TIMP3
APOC3
ATP5IF1
KCTD12
SCARB2
DPP4
HACL1
PHYH
SOAT1
NR1D2
SERPINF1
ZBTB16
INHBB
MGST3
SLC19A1
SAA1
SERPINA7
SLC6A9
SPHK1
TNFRSF11B
GCK
GPT2
SLC19A2
TMSB4X
GZMA

PCCA
SCD2
DACT2
VDAC1
ALDH3A2
BACE1
IL1RL1
ALAS2
FABP7
CES2
GLRX
ITPR3
MMP13
ALDOA
CRIM1
ECI2
PER1
NES
GAMT
MDK
ST3GAL5
ZBTB20
LY6D
DUSP5
DES
KPNA2
CREM
WNT5A
GPC1
PSRC1
COL6A2
RRM2B
SERTAD1
LRP5
TMT1A
KLF9
SYNPO
NHERF1
PAWR
LDHB
LSS
CDC25A
SLC27A1
RHOU
CDC25B
ECE1
EIF5A
IL7R
TMPRSS2
NUDT7
EGLN3

ENO2
RHOA
RXRG
TLE1
CAR2
PEMT
SACS
CXCL9
TFAP2A
ARHGDIB
COL4A2
UPP1
ETFB
ELF3
LBH
NUCB2
NCF2
DNAJB9
DNAJC3
KLHL24
CYB5R3
MFAP2
XRN2
GCH1
SPON1
ACKR3
AKT2
OLFM1
TOP1
NOLC1
RNASE4
SHC1
HBEGF
CYP8B1
SORD
MTHFD1L
EPHX2
FEN1
NRIP1
PIK3R1
TUBB5
CACYBP
TPI1
FMO1
ISG20
PPARGC1B
IDI1
LMNA
FCER1G
IREB2
NFKBIZ

TUBB4B
VCP
CAV2
CYP2C29
IKBKG
ABCD3
PCK2
TUBA4A
CSRP1
LAMP1
NGF
CES2C
ELK1
IL13RA1
BMP4
CPS1
ITGA1
IMPACT
THYN1
FGF21
BCHE
SERPINA6
APLP2
GSTM4
TUBB2B
ACHE
RND1
LIPC
ACE2
PPAT
PSEN2
ENPEP
CLIC4
CPE
ORM1
RELB
PDE4B
GJA4
QSOX1
RFC3
RPL10A
GPD1
HNRNPA1
SNAI2
LCN2
DNAJA1
EIF4E
FHL1
PTPRF
HPCAL1
TOMM20

BAG3
NFKBIB
PCSK9
RGS4
CADM1
OGA
TRAF2
MGST1
TCF7L2
TCF7
GTPBP4
SLC10A2
GATM
ARIH2
ANGPT1
OGDH
PSMB9
PSMC4
SRM
S100G
DHRS4
SESN3
ARPP19
ALDH1B1
LIG1
NCOR1
NR4A1
DGAT1
MAFB
TCF4
DUT
IFRD1
MYLK
CHEK2
CFI
FANCA
SOX2
DRAM1
NDRG2
NFIX
RETSAT
ERBB3
TFF1
FOXO3
PRTN3
SELENBP1
HSD11B1
SPTBN1
ANTXR1
APOC1
BCAR1

IMPDH2
LRG1
PKDCC
RNF13
SRGN
CYP2B9
MPDZ
LAMA5
SULT1C2
ACAT2
COL5A2
SELL
THBS2
CRYAB
RPL3
FGFR2
P4HA1
ROCK1
FADD
IL33
ARL4A
DFFB
MIF
CCN4
SEC63
KCNJ8
CYP2B2
LYN
CNN1
CYP3A2
PRNP
MAF
NOCT
CRIP2
EPS8
KPNB1
NPAS2
TRAF6
FAM107B
PTPRG
AOX1
DLK1
ATF2
FYN
LHX6
AEN
ITGB2
TMED3
GNE
KLF13
PAQR9

PXMP2
CXCL16
KDELR3
MAP2
NET1
PCSK6
CRY1
GPAT3
HNRNPD
MSH6
SERPING1
DSP
FCGR2B
GPC4
ICA1
PSMD12
PTPRD
TFAM
BRCA2
CDC42
MMP12
PLPP3
CD84
LIPE
NQO2
ATP6V0E2
ADHFE1
AKR7A3
HCK
MAPK11
PSMA1
SLC25A10
TSPO
SDHC
C1QB
CAPG
GBE1
IFI44
IFI27
MANF
TUBB6
PRKCE
BAAT
DDIT4L
SCPEP1
ABHD2
SMAD1
XAF1
BCL6B
ULK1
IFIT1

SMC4
ZMAT3
HK2
ARNT
UGT1A9
ATP5MC1
C6
FGL2
PRKCH
TTR
GCNT2
MATN2
GK
KLF10
SLC22A7
TNNI3
DHFR
MAP2K4
NIBAN1
MVD
TNFRSF10A
TNFRSF9
BBS2
TSPAN3
AMIGO2
KIF22
MAL
NFE2L1
PTMS
WSB1
ZMYND8
DDB2
HELLS
SESN1
ABCD2
PSMA7
HIPK2
NR2F2
SLC1A2
MBD2
HADHA
ATL2
HDAC6
MYBBP1A
PPM1K
FOXQ1
MKNK2
LTF
RBL2
TAT
MAP1LC3A

PRDX3
IFIT2
CREG1
KCNN4
MAPKAPK2
DLAT
HNRNPAB
HADHB
SPAG5
SPRR1A
PRSS23
MAOA
COL27A1
SMPD2
ITGA3
CD47
GPCPD1
LAMC2
PER3
ARRDC3
PLIN5
FNDC3B
ANPEP
ATAD2
PLD1
SLC51A
SMO
TRADD
NFIB
ST6GAL1
PEBP1
CFD
HBA1
TRA2A
WARS1
GAS1
HMGN2
LAMB3
OTC
QPCT
SLCO1A4
CLYBL
BLVRB
ANKRD12
E2F3
SLC37A4
TLR1
TPD52
NPDC1
ZEB1
GSTK1

MX2
SLC31A1
ATG12
DYNLL1
NOL3
OPTN
CLDN7
EPOR
FBXO30
GAS7
RASGRP3
THRA
AACS
CAPRIN1
CX3CL1
NFATC1
ID4
KRT5
LBP
PTK2B
RBPMS
ATP1B1
NID2
B4GALNT1
CHCHD10
CPD
NMT2
SPARCL1
CD83
IRF1
RRAD
SLC25A20
TGFBR1
GDA
HES6
SLC4A4
MX1
MTHFD2
NCOA2
MTR
TNFAIP8
ENPP3
KANK1
MCCC2
SLBP
PTGR1
AGTPBP1
SLC6A6
SMPDL3B
WSB2
LASP1

PROCR
PTMA
CTSK
TMEM47
DHCR7
TES
DDX21
MSRA
PC
SPRY1
PAM
TPP1
VWF
FZD4
ILK
LGALS8
EIF4A1
FOXA2
SORT1
COX8A
HBB
IL1R2
SLC48A1
TLE2
UQCR11
WNT4
ABCG8
CHORDC1
IFIT3
CARHSP1
ACOT7
FAM43A
MYH10
PRDX5
SLC11A2
SLC22A8
IDE
PAK1
SHCBP1
SUCLG2
TMSB10
MAP3K14
NEB
SFXN1
TRAF3
CDO1
DDX5
PLEC
SLC6A8
HYAL1
FLNA

ITGA6
AGTR1A
ALDOC
BMP7
CIDEA
LTBP4
HSD11B2
NR4A2
PPP1R3B
HNF1A
RAB30
TLR3
ARF1
GALM
RGS10
TNNI2
CYB561
CYP2A4
ADH7
CD52
CXXC5
GRINA
SATB1
ATG5
IL5
LAMC1
NT5DC2
SLC2A5
CFH
WFDC2
SLC38A2
SULT1E1
ACP3
ADGRL2
DIO1
MBD1
AVPR1A
CSTB
LGR4
ADIPOR2
ASPH
CALD1
DHRS3
HNRNPA2B1
PLOD2
PROS1
SDC1
SDF2L1
COL5A1
ALDH4A1
CDCA7

IRF6
NR4A3
ST3GAL1
NOS1
TNFRSF19
KLF2
FZD2
RAB31
SLC12A4
ITGA5
LRP1
ETFDH
GNG11
RPA3
AHNAK
VCL
SALL1
CBLB
TNFRSF11A
TMEM237
HADH
MYO1B
AKAP8
CD37
CYP2C9
LYSMD2
FZD1
LPXN
HAT1
XPA
ATP5F1A
BASP1
CIDEC
GSTT2
MYB
RGCC
UGT2B1
ISOC1
NDUFS8
MUC1
ARPC1B
HEY1
MTHFD1
TEK
ACOT4
PCOLCE
PTPN3
SIAH2
IL15RA
PPP2R2D
MAP3K1

BTG3
RAPGEF4
SLC7A2
SYNCRIP
FITM2
HACD2
MAGED1
RASD1
RCBTB2
CARS2
KNL1
PRIM1
TRAP1
PRL
CELA1
SLC28A2
ATF6
SMAD4
AKAP12
EMP1
ZFAND2A
ACADVL
HNMT
PTGDS
ROCK2
UGCG
CD86
MMD
SNX10
ETV4
CAVIN3
LAP3
PCCB
TIPIN
NOP56
SYN1
ABHD1
COMMD3
CAMK2D
CSRP2
INSR
LCP1
ADGRE5
APH1B
CFL1
FOXA3
NDUFS3
POLD4
ACSF2
CCT8
GALE

PKP2
PSMB3
SPSB1
ELOVL3
LPIN2
CORO1A
HDAC5
MFN1
SCARA5
CEACAM1
MCAM
MGST2
HAVCR1
HSD3B5
TUSC3
EFNB2
ALOX5AP
BCO1
CD63
DCT
GRB14
HIP1R
KAT2B
REPS1
RRAS2
SYT12
TAX1BP3
YBX3
SIRT3
FLT1
AGER
ALDH3A1
ATP2B1
NDUFS1
ITPR1
NAMPT
EPB41L2
MYO1D
NUDT4
PRKD3
HEXB
SLC1A5
PPP3CA
CKAP4
LSP1
MAPK13
OXCT1
STOM
SULF1
PGRMC1
CDH11

CLMN
EVI2A
FKBP1B
TUBA8
VAV2
SLC16A3
PINK1
BBS4
AQP9
JPT1
MYH6
SERPIND1
SLC46A3
YPEL3
SEMA3C
TNS1
STEAP4
FMO3
APOC2
GATA4
MDH1
PDIA6
UCP1
AIF1
ASGR1
SERINC3
STK38
TCEAL8
CLDN3
RPS2
TSKU
KLF12
SERPINF2
COL8A1
TNFRSF25
TPM2
LGALS3BP
TNFAIP2
EZR
MCM3
TSHB
MRC1
ACOT3
TPM4
PELI1
SIPA1L2
ADGRG1
F2RL1
ABCC9
CREBRF
H19

RAD23B
STARD4
TPD52L1
AP1S2
CES2A
ECHDC2
SGO2
SUSD2
ARHGAP24
OPLAH
PAPSS1
PLG
GOLM1
NKTR
CD24A
MARVELD3
AMACR
ETHE1
RPS3A
SET
CCL7
FGG
CCL6
COL12A1
FKBP1A
GPD2
MAP4K4
MT3
PODXL
RAC2
RPS6KA1
SMPD3
TGIF1
DVL1
RUNX1
SLC29A1
PDXK
AQP1
COX1
GLS
ATP5PO
COQ8A
DMGDH
DYRK3
MLPH
NECTIN3
SOX18
UBA7
VEGFC
ANP32E
IVNS1ABP

PCSK5
SOX17
TJP2
NR5A2
PTPRC
CTPS2
CYP2A1
DENND2B
KLK3
POLE2
CYP2C37
SERPINA12
ESRRA
WT1
DUSP3
LGR5
CMBL
SLC30A1
CES3
EIF3A
PLEK
SMAD6
LIF
ALDH18A1
DHRS9
WIPI1
TCF21
ENPP1
HMGA2
MMP10
SIGMAR1
APOA2
DCLK1
CCPG1
CD53
FXYD5
NTN1
TTC3
TEF
N4BP2L1
USP10
CCNE2
CCT3
NRAS
PPP1R10
EIF3J
LRP11
PGF
GEM
IGFBP7
SCAPER

TNS2
LMNB1
ALDH9A1
BLNK
SLC38A3
SYNJ2
POMC
YWHAB
FBN1
ITGB3
C8A
PLEK2
RDH11
SDS
TAP2
ANG
MAP3K8
TMEM97
MRPL49
TKT
CCR2
CEMIP2
GNL3
P4HA2
SLC13A3
VEGFB
EIF2A
CCNC
CORO6
ODF2
PTGFRN
AHSG
PSMB10
BZW1
PDGFC
RAPH1
TLR6
PNP
IL17RB
LPCAT3
ABAT
EP300
DUSP8
FRZB
METRNL
NGEF
PSMB1
RPLP0
RPS20
SMPD1
BDH2

CASP2
COMTD1
DIO3
FMO4
MLLT6
MOGAT1
MTMR7
NFAT5
PIK3R3
PLA2G6
RAD50
SOX13
USPL1
ZHX3
MAN2B1
HSD17B2
DEPTOR
HSPA1L
NASP
RPS19
ABCE1
CD82
FASL
IRF8
PPRC1
CKAP2
FAH
OPA1
CSE1L
CSNK1A1
EMP2
RGN
ADAM11
EIF2B3
MPEG1
NDUFB2
PXMP4
SYT7
CS
FOSL2
RAN
SLC7A7
ADSS1
GSPT1
SDHB
MACF1
OLFML3
PLAGL1
PSIP1
STX3
TFDP1

HMGA1
CLTC
CRY2
TCP11L2
BMP6
SLC22A2
GLDC
GOT2
IFNGR1
MSN
UNC5B
VTN
DHRS7
FABP2
RPL23
YWHAZ
NUDT1
CA3
LONRF3
PAK2
SH3YL1
TMEM98
TWF2
RORC
RRP12
SGMS1
TCEA3
IDH2
ELOVL2
OGT
HTRA1
NPPA
SLC2A3
ACTG2
GLS2
INCENP
PTPRK
SMARCA2
CAPN1
DKK1
IL12A
BMP5
DECR2
FUT8
FZD8
HMGN1
MAPRE3
WLS
XPOT
FIGNL1
MTTP

PTPN1
CYP2C11
FKBP4
LTB
BHLHE41
CSAD
CYFIP2
MFN2
LAPTM4B
CIRBP
PDE5A
USP18
FTL1
RNPEP
IER5
IFITM3
RBM3
TSC2
TIMM10B
ACAD9
ACSS1
TLN1
UBE2L6
COL2A1
ACADS
PPP3CC
RACK1
MYL9
SRSF7
TM7SF2
BCAT2
BLVRA
CENPK
CITED4
CLGN
ERO1B
GNPDA1
ATP6AP2
MID1IP1
TRPM4
ABCA3
APCDD1
PLP2
AIFM2
DKC1
RPS3
TGFB1I1
ABL1
CYP51
EGR3
ANKRD1

DLD
ELOVL5
GJB2
CPQ
DCK
SELP
TPMT
ARHGAP5
SH3BGRL
BCAT1
JUP
ANP32A
CELF2
ACER2
CHST11
CTSE
HMGN3
ARHGDIA
CCT2
FBXO5
HNRNPH1
PGS1
PSAP
CRADD
ZFPM1
ITGA2
SFPQ
BBOX1
CASP10
CDA
PRSS8
STAB1
STARD5
TMEM86A
PSMC5
REEP6
ASRGL1
BMI1
CORO2A
CSTA
NIFK
ORM2
OSBPL3
NCOA1
CCL4
FAM107A
HSPB7
MPP1
SLC43A1
ACSL3
GRB2

GSDME
HIGD1A
PTN
RGS5
WNT3A
ACP5
PRKAA2
PTX3
CD24
CTPS1
STC1
AGPAT2
ARHGEF3
PERP
PLIN4
RAD51C
STK39
TRIB2
CBX1
SLC66A3
TFPI
NUMB
PRKCI
ADORA2A
NCOA4
PDZK1
PRDX4
PRKDC
SC5D
ACOX2
COL14A1
E2F2
ENDOG
FLNB
GATA6
HAS2
TPR
TWIST1
BNIP3L
C5
ITIH4
MMP8
OSBPL1A
PRKCQ
RPL28
LRRC28
DHTKD1
LACTB
DBN1
KLF15
PPM1D

STAT2
TCP1
ADAMTS4
CLOCK
ELF1
FBLN2
HSPA12A
IMPA2
NDRG4
PSMA4
RAB8B
SLC13A5
UBE2H
VASP
CXCL5
TXN
B4GALT5
CHPT1
HNRNPK
ANK3
DUSP2
MYOF
CRYL1
EEF1G
GSTT3
PABPC1
PANX1
PBX3
PPM1G
RIDA
EBPL
PNRC2
SLC38A6
GFOD1
PAFAH1B3
PSMC3IP
ITSN1
STEAP3
CISH
ERRFI1
GARS1
SYP
ACTG1
NEDD4L
CCL11
MERTK
EEF2
MAOB
NARF
HPD
MYCBP2

NUP153
ADORA2B
ALDH6A1
TXN1
ASAH2
BLCAP
MUG1
TM6SF1
ABLIM1
APOD
PA2G4
IFI35
LIN7A
LPO
SSR3
TFF3
TOB1
CMPK2
IRF9
NME2
RBL1
RRS1
SH3BP5
TKFC
LECT2
DUSP4
IGFBP6
IRF3
PLS3
CD151
PLAAT3
SSB
SYTL2
CPEB1
CSTF3
MYO18A
SMS
KLF11
KLHL21
RHBDF2
RHOBTB1
SLC25A13
CES1
CCT5
HILPDA
LGALS4
PLA2G12A
PLCG1
TARDBP
HYOU1
TUBA1A

AKR1B7
ANGPTL2
C8B
MRPS6
PARD3
RASL11A
CTSA
NFE2L3
RRAS
CYP2C55
EIF4EBP2
IFNAR2
AKR1B8
IFI6
IL7
ACADSB
TBX6
TMEM245
AGL
CABLES1
CD4
NFYB
SLC2A6
SPINK1
TDO2
TRAF1
FGF12
NDUFS5
PLXNB1
PRPF8
RASGRP2
UTP20
DBP
FAM171A1
PFKFB3
TPK1
HDC
MAP1B
DKK2
FASTK
LRATD1
POPDC2
TTF2
CRK
MYO5A
PSMC3
SLCO1B2
TBC1D4
WWOX
NPC2
PSMA2

RPA1
FADS3
HEXA
SLC27A5
CXCL3
LIMA1
RPL6
BCL2A1
LAMA3
NOTCH2
COX6A1
FMNL2
HIVEP3
PITPNC1
PTER
TMEM176B
ELP4
GFI1
MLH1
ESPL1
FBN2
GFRA1
ABI3BP
ADAM15
KHK
MEG3
NLRP12
RALGDS
SNRPD1
PRKACA
STS
SPTAN1
NPY
APLP1
GPLD1
NIN
RASSF2
TRIP12
UQCRFS1
FETUB
OSMR
TRIM2
VEGFD
C1QA
PDLIM7
TGM1
ATP6V1D
CLTB
ITGAX
NBN
RAP1GAP

HPX
FGFR3
AIG1
PTPRE
TM4SF4
ABCA6
PCX
ARSA
CYP2D22
SPEG
TLCD4
AARS1
BAMBI
CYP2A5
HHEX
KIF20B
SRRM2
MEF2C
PSMB8
AAAS
AKAP9
ATP6V0B
CLPX
DHX9
FZD7
GRK5
LACTB2
NDUFB6
POLR1B
PRRG4
SYNGR1
TFCP2L1
TTC39C
VCAN
FGB
ACAA1
CCL8
CD48
HEATR1
PSMD7
RABGAP1L
SERPINB1
SRI
ST3GAL6
DDX17
IL15
CLCF1
ELAVL1
HBP1
HSD17B6
ITGB6

GRPEL2
TNNT3
ZDHHC23
B4GALT6
DAPK2
EPN2
HOOK3
GPI
IFITM2
KMO
USP7
DCUN1D3
ABHD6
BAIAP2L1
FCGR3
IL13
RHPN2
SLC25A29
TSHZ1
MBNL2
SMARCA4
ABHD5
ADAMTS9
DLL4
DMBT1
FAM20C
MIR34A
PCYT2
BARD1
CD55
GLIPR1
IFIH1
MTERF2
KITLG
SLC16A7
VKORC1
ENO3
PLA2G7
SOX11
TRAM2
HAAO
PBXIP1
RIN2
STK4
WIF1
LOXL2
MTUS1
CPNE8
EMP3
FGL1
RPL4

ZFHX3
LAMTOR5
MPHOSPH10
NEDD1
PAQR7
AASS
CTNNAL1
HOPX
HSD17B11
LAPTM5
RRAGD
MB
STAT5A
NCAM1
RHOC
ADRB3
FSCN1
NCAPD2
HLA-DRA
FLI1
LPGAT1
PCYT1A
PFKFB1
RASSF5
TLR5
TMEM176A
ABHD14B
LHFPL2
DACT1
NAV1
PYCR1
STARD13
ACTN1
CDC42EP5
FGFR4
NFKBIE
PGM3
PSMD1
PTGIS
EPO
HNRNPDL
NABP1
COQ9
FGFR1OP2
USP8
AKR1C3
HK1
CADPS2
MAPK7
NELL2
PTPN12

RBMS1
RDH10
RHBDF1
VIL1
BMP1
CBX4
MRPS18B
PTPRO
SRSF11
YPEL5
BCKDHA
CTSF
IL6RA
TMEM140
ADAMTS5
PMVK
WWTR1
CYP3A7
LAMP2
PFN1
RIGI
SLC1A4
FARS2
FCMR
SCCPDH
ATP7B
AKR1C1
GNAI3
MBL2
PLXNC1
PTBP1
PXDN
STK10
TAPBP
DIAPH3
EHD2
FAM13A
HPSE
IL10RB
PCM1
PTPN21
SMIM14
XRCC3
CAMK2A
HSD17B3
ITGA4
CTNNA1
EIF1
EIF4G1
LRRFIP1
MTSS1

PFKM
PSMB5
BMP2K
CDC27
E2F5
RAB3D
SLC43A3
SMYD3
TDG
TRP53INP1
IRAK1
CD27
E2F4
MCOLN2
WDR36
GM2A
LYZ
MPST
SEMA7A
TNNC1
TRA2B
CYGB
PID1
ERMP1
F8
REV1
RHOBTB3
TLE3
UBA52
DHX33
NEU2
RBP2
CCL20
XPC
ADAMTS7
PDZK1IP1
PMM1
PTGR2
COX4I1
DGKE
PML
AKAP1
CCDC80
CREB5
CYP4A1
RIPK2
SLC7A8
CA12
CRISPLD2
EMB
LHFPL6

NRF1
AATK
BEX1
PRKAG1
RASA4
RNF6
RTKN
COX6C
EBNA1BP2
PLAA
QKI
RNF125
ST8SIA4
TFE3
RIPK1
FPGS
PARP12
PRKAR1B
TINF2
ADCY7
AGTR2
DDX39A
SLC5A3
SVIL
SYNE1
YBX1
ARL4C
PAFAH1B1
RFTN1
S1PR3
SLC23A2
IL6R
ADAMTS2
DAAM1
LAD1
PSPC1
SEC23B
SPTLC2
TRIM16
UBE3A
ZAP70
MDFIC
PKD1
PSTPIP1
EIF2S2
F7
GINS2
CBR3
GLUD1
OLR1
CD81

PIK3CB
RELN
ADAM10
CDKN2D
CMTM3
MAMDC2
MUTYH
RECK
TBC1D31
TCN2
WDR3
FOXO4
GNA14
NPC1
PPM1A
RPL22L1
SCARA3
SKAP2
ATP1A2
LIPG
YWHAQ
ZYX
DMTF1
ISLR
LIN7C
SETD1B
UGT2B7
ATP10D
BEX4
CBFA2T3
CILP
COL4A4
FBXO31
NR2C1
S100A16
TBL1XR1
CPN1
ECHDC1
ENTPD2
MBD4
NUP58
SMARCA5
TIMM9
FAAH
AGRN
RPL13
ACAD11
APOA5
BAZ1A
CGREF1
ERCC4

EXOSC8
FBP2
FLOT1
FNBP4
IL2RG
METAP2
PYGB
RFC5
SHISA2
TSPAN8
ABCC8
HMBS
PSMB7
SARDH
TP53I3
GC
PGM1
SULT1B1
ATP13A3
NMB
PFDN4
RASA2
WDR54
ATP11C
CUL4A
DAO
H1-0
LIMK2
NLE1
RFC1
RFFL
TSPAN13
ADRB2
ALDH1L1
BCLAF1
TIA1
CCN5
CD8A
GPSM2
MAP2K5
MPZL1
RAD18
RPL7A
CD80
SCNN1A
EFEMP1
SLC8A1
COX5B
EDEM1
EHD4
ILF3

MFAP4
P2RY2
RBBP8
RGS1
SAA3
VMP1
YARS1
NPPB
VAV3
XPO1
GREB1
ILF2
MLF1
MRPL17
PTPN18
SPSB4
ANXA8
CAPZB
OXR1
PGM5
PKIB
PRMT3
TPST1
ATP1A1
CDC73
KRT4
LPAR6
PARP9
PPIL3
PRF1
REEP5
RHOBTB2
RPL12
ASL
PPP1CA
RNF19B
ABI2
CASKIN2
DNAI1
HSD17B7
EIF4B
LPP
NTRK1
THRB
ASAH1
LCK
LTBP1
DLGAP4
ELK3
ITPKA
MADD

NLN
RCL1
TMTC4
ACY1
AJUBA
ANGPTL3
BCL2L2
CORO1C
NDUFAB1
OAF
SLK
CYP3A25
AGO4
CNKSR3
PPP2R5A
SLC35G1
FILIP1L
HP
SPDL1
CDK5R1
ETV5
FDXR
GAA
YWHAG
MRPL33
NFS1
PCBP3
PLA2G1B
ZNRF1
ACTR6
PI4KB
ATR
DYRK2
EPB41
F10
LGALSL
MIA2
PTPRN
TACC1
TRIM28
WNT11
YY1
ABCF1
C1R
PRXL2A
RNF144B
SLIT3
GREM2
PAK1IP1
PCOLCE2
SKI

SLC25A22
SSX2IP
ORM3
E2F7
GART
RRBP1
ZFP36L2
COX6B2
MAP4K1
MIOX
MORF4L2
RGS17
ST3GAL2
ST3GAL4
AQP4
IL6ST
MIR21
AQP2
TMEM63A
APOM
BCL10
CHAF1A
CNN3
IFI30
RHEB
SLC16A10
CSF2RB
MPZL2
RPL32
SLC13A2
ESD
PLXNA2
UQCRC2
CES1D
GRAMD2B
NME4
NUAK1
PRPS1
RAMP2
SLC23A1
STAU2
SUCLA2
UBA5
RPP40
TBC1D10A
APPL1
CYP24A1
ETNK2
OTUB2
PPP1R18
SRPK2

ORC6
OXTR
PDE3B
SLCO4A1
TENT5C
TP73
ATOH8
AZIN1
EEPD1
MYO1E
NEXN
NFATC3
PLEKHF1
PTHLH
SLC39A14
ST13
TCF12
WIPF3
RNF122
APPL2
CCDC92
DNAJA4
EPHA1
GCGR
NBL1
CRABP2
EIF4A2
PROC
SMC1A
CRYM
FOLR2
MICAL2
SH3D19
ATP1B3
CCNDBP1
CEP135
EPB41L5
NINJ2
NOG
SH3PXD2A
SUCNR1
TBX21
TSPYL4
UBR1
CFTR
AVPI1
DARS1
DTX4
GCAT
GSTO2
IER5L

RCN2
CES1C
PROK2
REEP2
NNT
PF4
GPER1
IDH3A
SLC47A1
ABHD4
NINJ1
SDCBP
CD2AP
COQ10B
HERC3
HKDC1
IPO4
MCCC1
MEGF6
NUAK2
RNF4
SLC35D1
SSBP3
TIAL1
TOX
FKBPL
BCAR3
CAD
ELN
FBLN5
RETN
H4C3
PLCD1
RAD54B
SORBS3
TMED2
WIPF1
GSTM6
MTIF2
PKN2
SLC25A28
MAP3K7
PSMC2
RPL17
STK17B
AP3S1
MDM1
MPHOSPH6
POLD2
RAB43
SCHIP1

SERPINA3K
SEZ6
VSIR
DBT
FH
FNDC5
GAB1
NAB2
NSD2
RHOQ
SEL1L3
SLC1A1
SSR1
TENM3
USP14
ALDOB
EBP
NPR3
SOCS1
CDH6
CLEC2D
CYSTM1
DGKH
FMO2
RERG
SAP18
SSRP1
THNSL2
C1QC
CNDP2
PDK2
PTPN6
SLC34A2
ABCA5
DDX46
HERC6
IRF5
KLF3
NKX3-1
PRSS12
RAB14
RPS18
TIMM8B
ADAM19
CDC14B
MR1
PPFIBP1
PRDM2
PTPN13
RCN1
RUVBL1

NRCAM
SERPINC1
MTF2
RRP1
TMEM184C
TPRKB
ADAMTS15
AMOT
CLPTM1
EXTL1
LY6A
NETO2
NOB1
SAC3D1
SETD4
ADPGK
CCT6A
CD3D
CD59
CDS2
CHAC2
COL7A1
CSNK2B
CYB5B
EMILIN2
GPX7
MEOX2
PRODH
TCERG1
PDE4D
ADAM12
C3AR1
DAD1
FNBP1L
FXN
GNA12
MSX1
NISCH
RAD51B
RPS25
SSBP2
FSHR
PRDM1
VDAC2
MEST
NCAPG2
NEK6
NUCKS1
PDE7B
PSMD14
RAI14

RBBP6
SMOC2
MTHFR
F13A1
CYP4B1
FIS1
PMEPA1
CACNA2D2
DMXL1
NDUFS2
SLC8B1
ANAPC1
EIF2B4
HCFC1
INHBC
MARVELD1
MIR22
NDUFAF4
PPP1R15B
WAS
GREM1
GUCY1A1
BIN1
CDV3
CTBP2
GPR155
MICAL1
MTAP
NDUFA5
TNFAIP6
TPP2
EXT2
TMEM86B
TPT1
UBE2I
FOXP1
PGAM1
CBX7
CRABP1
ETNPPL
FCGRT
HOMER2
IKZF1
ITGB7
LYZ2
MAP3K4
PABPN1
PANX2
PDGFD
PPA2
RAD21

RPF2
SGK3
TK2
MYO1F
SCLY
SNX24
TCEAL9
TGIF2
TMEM14A
CYP2R1
FAM162A
HSPA13
PBX1
PRMT5
EEF2K
ESCO2
HAUS6
KATNAL1
PDPK1
PYGM
SLC25A34
SLC41A3
TBXAS1
APRT
BAG2
COX6A2
DDR1
EI24
HIBCH
LDHD
ME3
PRPF19
RALA
RPS27
TJP3
AK3
ANGPT2
BOK
CISD1
DDB1
GALNT16
HDAC4
HNRNPA3
NPEPPS
SLA
STT3A
DPYSL3
HSD17B10
MAPK10
STK11
TP63

FCNA
CIDEB
HDHD3
LCORL
LRATD2
LRRC20
MTMR11
NAT8L
OLIG2
TAF9
UCHL5
FES
MTMR6
RCHY1
SLC25A42
TSTD1
VRK2
SLC12A2
AK2
DGKA
GGH
H6PD
HIVEP2
LAMB2
MYH11
POLA2
PPT1
RASSF1
SH3KBP1
SLC22A4
XRCC6
APOH
CRTAP
EIF4G2
ELOVL1
ENTPD1
FJX1
MYL1
RASL11B
SORL1
CD302
CDK2AP2
FAM114A1
HEY2
PAPOLA
REV3L
SERPINB1A
SLC16A5
XPNPEP2
PRPF4B
UBAC1

CDK8
CERS5
CREBZF
DYNLT3
MYL2
RNF14
S1PR5
TXLNA
NR3C2
TIPARP
CEBPG
YWHAH
CLK1
SLC16A6
TARS1
CXADR
JADE1
PRKAG2
UAP1L1
UTRN
GNAI2
CTSZ
HNRNPF
LMAN1
NXF1
PLXDC2
SEMA3B
SON
CALM1
CCDC50
CLEC4D
DSE
RSPO1
UBE2G2
F12
FKBP14
H1F0
IGSF3
RPS4X
SLC24A3
TAX1BP1
WDR26
ZCCHC24
AKT3
FGA
LAMA1
PFKL
POLD1
RSRP1
SLC51B
UQCRC1

CAMK2N1
PAN2
POLE3
RBM19
RSL24D1
SETD7
SF3B3
NCBP2
AGXT
ALDH8A1
ERCC5
EWSR1
FRMD6
LGALS9
PRDM16
SUCLG1
WWC1
ADRA1B
CTTN
PAX6
SRSF5
CYP2A6
FBH1
ANP32B
APLNR
BORA
BUB3
CCAR1
DNAJB11
DROSHA
FOXP3
IFI27L2B
IFRD2
LIMS1
MDC1
MYOM1
NCOA6
NRBP2
OSBPL6
PDCD11
PDHA1
PLEKHA5
PPIF
UOX
TXN2
ATOSA
BEX3
BRD3
EPB41L4A
FZD3
GATA2

GPX8
LATS2
MLKL
NDUFA10
PGM2L1
PLXNB2
RAD23A
RASA1
RETREG1
RGS19
RIF1
SLC33A1
SMTN
TBC1D9
REL
S100P
SYK
AGO2
LTA4H
ANKMY2
CCDC28B
SLC49A4
ABCA8A
EEF1E1
HPN
MMP11
PRELP
SRR
TMTC2
CDK5
JAK3
SAMHD1
SLC25A5
CDH5
MITF
SLC22A3
BTBD3
CHCHD2
CREB3
MSC
PGLYRP1
PHC2
PRPF6
SENP7
SLC14A2
SULT1D1
ADGRV1
AMD1
CHN2
HSD17B12
IL13RA2

LAMA2
MAPRE1
SERINC2
UPB1
CAST
CCL9
ARID4A
ARMCX3
CHD1
GDPD5
MAN2B2
RASD2
SUV39H2
GSDMD
TUBB3
ANTXR2
CYP4F14
MAP4
SQOR
ATP11A
DPYS
PDX1
PIK3AP1
RRAGC
VAV1
TMEM115
PIK3CD
ETFBKMT
PCSK4
TM2D2
TTL
ZCCHC14
ANXA7
ATP6V0D2
DUSP16
PNN
SERPINB5
UBE2D3
RET
DEXI
METTL9
RNF144A
SNCB
TMEM43
BACH1
CBX5
EIF2AK2
H2BC21
IARS1
LPAR1
SEMA6D

CETN3
CLEC10A
COPG1
EIF4E2
EIF4H
LAT
NRTN
RANBP2
SAR1A
SART1
SLC25A51
EFNA5
HSD3B7
IGF2BP2
KHDRBS1
PPP2R2A
PPP5C
ALDH1L2
INPP5D
PDLIM5
CLEC7A
DDX1
FOXN3
GCNT3
ITIH2
MAD1L1
SIRPA
TET2
DEPP1
CSK
PANK1
GEMIN2
LRRC39
PNLIPRP1
RAB26
RNF141
WBP4
AKR1C14
ARHGAP25
BOC
CAR8
COQ10A
FUCA2
GLT8D1
MFNG
NRP2
PATL1
PELI2
PLEKHA1
TTPAL
TUBB

WFDC1
ZBTB40
BSCL2
CBX6
CDK18
CEP57
DDHD1
FANCC
H2BC5
PDCD5
PIF1
SLC45A3
SPEN
SURF4
TMBIM6
EDNRA
ADGRG2
BCKDHB
GGCT
HMCN1
HTRA3
PABPC4
PGRMC2
PTPRU
RPL34
SLC37A2
SOX6
TIMM17A
ARG2
CHRNA4
ARHGAP18
GFPT2
HDLBP
MEOX1
PRKAR2A
TACC2
MSR1
NCOA7
SLC38A1
TSHR
DLG1
PAH
LHCGR
ASF1A
CYP2F2
GABPB1
GCA
MYO1C
NIPSNAP1
PEX19
PILRA

PLPP2
PTPRM
SNAP23
STXBP6
TCIRG1
TCOF1
THRAP3
USP15
CANX
DERL1
HRG
IGSF6
KRTCAP2
LIG3
NUDT21
SP110
OAS1
PIM3
RPL8
SEC61A1
ABHD14A
FOXRED2
KISS1
NFYC
NKAIN1
PDE4DIP
ACSM3
SPRY4
CYP2C38
RNF169
TRIT1
AMPD3
SLC7A1
EEF1B2
EPB41L3
KRT15
NDUFS4
NTN4
P2RX4
PNPLA8
RBPJ
SKIL
TRIO
CALML4
HNRNPR
MSX2
MYO6
NIT1
SLC25A33
TMED10
TMEM158

UBE4B
BST1
CAMP
CLSTN2
IGHM
IRX3
KITL
LRIG3
LRRC8D
MBNL3
MPP7
NLRC5
PCDH1
PTBP2
SCN8A
SPG7
TMEM135
UNC93B1
MXD1
ACYP1
CBLL1
IMPDH1
NRARP
OSBPL5
SNTG2
TMEM14C
ITGB4
ADAM23
ANKS4B
MFSD1
MOK
SEM1
TMEM132A
CRYZ
HNRNPC
PPP1CB
CCR7
EFHD1
EFHD2
ITGA8
NEBL
PPIA
PRIM2
RSL1D1
ERCC8
MEIS3
RNASEH1
EPHA2
GPHN
HMGCL
INF2

LYAR
MEIS1
NR2F6
PDP2
PSMD4
RIPOR2
SPATA13
VHL
AHCYL1
B3GALNT1
CDCA7L
CUX2
EXOC4
EZH1
MCOLN1
MPZ
PIM2
PITPNM1
PTP4A3
RIPK4
RNF103
TOR1A
CERT1
CHD1L
CLEC11A
CLUH
DPAGT1
EGFL6
MAPK4
MGAT5
MRPL18
NUBPL
PLCD4
PSTPIP2
RIOK2
RNPS1
LY96
SLC1A3
LRP2
NCEH1
TM4SF1
EPB41L1
MAPKAPK3
TAF1D
ARHGAP8
BTF3
CAMK2G
CHFR
COPB2
HSPBAP1
HTT

KAZALD1
MAGI1
NUDT6
PHEX
QDPR
RBMS3
SHISA5
TFR2
TNKS1BP1
TSPAN4
VEPH1
WNT10B
PTPN11
F5
ALPK1
ARSG
ATP2B4
BMPR1A
DDX10
FLCN
FXYD3
HACD4
MANBA
MMP16
PGPEP1
RTN4R
SERTAD2
SLC5A1
ARHGEF2
CSF3
DTYMK
PLCE1
ATG16L2
BLMH
CHDH
CPED1
CRYAA
DEF6
EPDR1
FPR1
H3F3B
KHDRBS3
MRAP
MS4A1
NT5C2
RSF1
RTN2
ST7
TRAF3IP2
TSPAN33
ATG3

GALNT10
GNG7
HGD
MAGED2
PDP1
RNASEH2A
RPS21
TNRC18
AHRR
CLIC1
NEAT1
PTGER3
KRIT1
PDE1B
PPDPF
SAP30
CRYBB3
CYP2C54
APBB1IP
CSGALNACT1
EVA1A
NIP7
SLC9A3
SNRPF
TSPAN5
TYRO3
RIPK3
RPA2
RPS15
UGP2
ACYP2
ASPA
COL17A1
ERP27
G2E3
GALNT15
LACC1
NADK
OS9
PPIC
RAP1GAP2
SFTPB
TCF3
THBS4
ZMIZ1
AMBP
RDX
ANKRD11
CEMIP
COLEC12
COX17

EEF1A2
ITGA7
OAS2
BANF1
CHSY1
GAB2
GSTP2
KLHL5
LUC7L
PLPP1
PPIL1
S100A13
SERP1
SLC4A11
SRPRB
TNNC2
TRMT1
TTLL7
UQCRB
WDR12
XRCC4
GNG4
IRAG2
MTERF3
SUGP2
TMEM165
ABCB6
ARRDC4
GLI2
NR2F1
ADAM9
DNAJC2
EIF3C
EVL
GLA
SLC39A4
ZC3H12A
BCCIP
BYSL
ECHDC3
EEIG1
EEIG2
G6PC3
GALT
GOLIM4
HOXD8
LARP6
LZIC
MBD3
NEO1
RCAN3

REG3B
WDR75
NCOA3
RPS15A
SYNM
ANO10
CDH17
COL11A2
COL4A5
FEZ2
HAGH
JMY
MLYCD
MYEF2
PHLDB1
PSMD13
SH3BP2
SPA17
UGT2A3
ANKRD40
CD300LF
CYP4A12A
GSPT2
MMADHC
MYCN
RANBP1
CCN3
FXR1
GBP4
KALRN
CXCR2
ALG14
CPT1C
DENND2C
IL11
MMD2
S100B
SCX
TMEM150C
ACOT12
CCRL2
COLQ
FAM110C
MARCHF6
SLC25A19
ZDHHC14
C4B
RFC4
SLC38A4
ADGRL1
ATXN2

DCUN1D4
FABP6
HR
PDZRN3
SELENOM
SERINC1
SRRM1
TIFA
CLDN5
NGFR
ADCY6
ALOX12
ANXA11
CAPNS1
IFNGR2
MACROD1
NFE2
PDZD2
PLD3
PRKD1
RPS16
UBE2N
OTUD5
ADAM8
DFFA
GBP1
A1CF
DCAF5
ELFN1
NEK4
OGFOD1
TAF5
ZRSR2
SRSF1
LMBRD1
RBBP9
SNHG11
STXBP5
ACBD5
AGFG2
CACNB3
CRYBG1
EIF5A2
ERH
GPC6
H2BC14
NEK7
NNAT
PLEKHG3
PNPO
PRR13

SOCS5
TBCB
WDR4
C2CD2
LY86
MST1
P2RY6
PIPOX
POLD3
RTN4RL1
SH3GLB1
TRAF5
ZFAND5
CIITA
LRP6
OASL
PSMA6
SGK2
SMPDL3A
KDM6B
RPL15
NARS1
PSMB4
IL2RA
AGPS
BMS1
CDC16
CDC42BPA
CHCHD3
CILP2
CYP4A8
DEDD
DNAJB5
FXYD6
INS2
ITPA
LRPAP1
MAP3K12
MIB1
NFX1
NFYA
NMT1
NOC3L
NTHL1
PACSIN2
PARP16
PCDH17
PDCD2
PRG4
RAI2
RAPGEF5

RWDD2A
SLC39A1
SLC43A2
SOX8
TFAP2E
TNIP2
TSPAN17
WWC2
DAP
DNAJC10
FLT4
RPS13
SMC3
TET1
ARNT2
GPRC5A
IVD
FECH
KRT17
MYH9
RPS27L
P2RX7
GRIN1
DBF4
NRXN1
POLE
PPP2R2B
ARSB
CDK7
DNAJC12
DNM1
PDPN
PEA15
RPS14
RUNX1T1
ABI1
ABTB2
AIMP1
CERS6
CLSTN3
COBL
DUSP14
GHITM
GTF2IRD1
KCTD15
PPFIBP2
TMEM45B
TMOD1
TRAFD1
ALDH1A3
CNR1

DYNLL2
EYA4
FOXP4
HOXB9
IL18R1
MICALL1
SLC16A4
UBR2
UPF3B
VPS13B
ZPR1
CABIN1
GOLPH3
KLHDC2
LST1
NUFIP2
PHKA2
ROGDI
SH3RF2
SKIC8
STX2
TRIM37
ENTPD3
HPS4
PLSCR2
SLC22A17
ST6GALNAC6
UBQLN4
UGT1A2
ECHS1
FLNC
DZIP1L
FAN1
HMCN2
CALM2
EHMT2
IP6K2
LRIG1
OIP5
PCP4L1
PIGA
SFXN2
SLCO3A1
ZDHHC2
BCAM
SIPA1L1
SMAD5
STAMBPL1
ZCCHC7
ZWILCH
CDKAL1

CDR2
FKBP10
GIPC2
HTATSF1
HYCC2
ITGB3BP
KIF3A
LYPD1
OGFR
PPM1L
SELENOI
SH2D4A
SLC39A11
TRAK2
ULK2
COX2
BLM
NPHS1
ABCA7
ABHD15
ARHGAP10
ATN1
BPNT2
C4BPB
CBX2
CCNL1
FBXO21
GFER
LMO7
PFKFB4
RAD52
RARG
RASAL1
RPRM
TFEB
UGGT2
ZMYND11
ZRANB3
AOC3
MLLT11
MYADM
NAA15
SORBS2
STEAP1
CUL5
IL18BP
OSBPL7
ACOT9
CAPN5
CREB3L2
KDM7A

MDN1
OGFRL1
TNNT1
AGTR1
AKR1D1
C1QBP
GOLGA8A
GTF2E2
ATP2A1
CMIP
COPE
EPS15
FGD6
FTSJ3
HMGN5
MASTL
NAGK
RAB11A
RENBP
RYBP
SPINT1
MYH7
DLG4
IL23A
BCL11A
CNN2
CYC1
HCAR2
MAL2
PPP2CB
RPS6KA2
SLC25A1
SYT1
TRPV6
BLOC1S2
CHKB
CLDN14
DLG3
FAM117A
SAT2
SLC22A15
SMC6
ATP6V0D1
DOCK10
GPRC5B
GRHL1
ITIH1
KLF7
KPNA4
MALT1
NBR1

PDLIM3
PROX1
PTPRN2
PUS1
TNFAIP8L1
UBE2B
LRP8
LTBP2
PLIN3
LAG3
RAB24
SEC23IP
AGPAT4
CLIC5
DAGLB
EPHB6
NDUFA7
PTPRB
RPL39
TCF7L1
TIMM10
UBE2S
MLEC
PRKAB1
PSMC6
RPS24
YWHAE
ANKRD13B
CCDC120
DYNC2LI1
FAM234B
IZUMO4
RPE
STX4
CTHRC1
CYP27B1
GLG1
ME2
PHIP
PLEKHB1
PRPS2
SP3
SPINT2
SPRED1
TANK
ACAA1A
AGAP2
CACNB1
CDC42SE1
CPXM1
FBXL7

HERC1
KSR1
MANEA
NPHS2
STRN
SYCP3
TMEM62
TRIM35
TWIST2
YIPF5
YLPM1
NEFL
ARHGAP26
GULP1
LBR
MCF2L
MRPL12
PCYOX1
TENT5A
UAP1
ARAF
B3GNT5
CDH4
HOXA5
KAZN
LEAP2
MAGOH
OTUD1
PKIA
PLCD3
PNKD
SIX1
SLC44A4
SRPK1
SRPX2
STX12
CD2
EMC9
FMN2
HM13
MIR200B
MRPL24
NUP133
PPP1R1B
RUFY1
SDK2
SLC5A9
SULT1C1
TBX3
TIMM21
ZC3H8

ANKRD37
FERMT2
LMCD1
ATP1B2
CREB3L3
DNAJC9
DUOX2
ENDOD1
ITIH5
OGN
SLC25A23
TNFSF9
ACAA1B
ART3
ASGR2
BTC
CD46
CLP1
GLTP
MATK
MIX23
NCKAP1L
PCDH10
PES1
SEMA5B
TMEM64
UTP14A
VASH1
FHIT
IL2RB
LTBR
PLA1A
ANGEL2
CLDN9
KCNA3
TESK2
ADSL
ALDH1A2
HAO1
MAP1A
NSG1
RNF24
SMARCD2
ZBP1
ADI1
FGR
LRBA
LRP4
ZFHX4
CNNM4
MBOAT2

OAZ1
ORMDL2
RELT
ROS1
RPS6KC1
STK24
EBF1
NPR2
SEC31A
ADK
GABARAPL1
HDAC3
APBB3
CMTM6
HAND2
KLB
RBM26
TULP4
PRKACB
ACNAT2
CES4A
AUTS2
CLCN4
COQ3
GBP3
LPCAT2
RBBP7
RBM24
SEPHS1
ATP6V0A1
CARMIL1
CIT
COX3
S100A1
SLC39A6
SLC6A12
ABLIM3
ANO1
CUX1
FKBP11
KNTC1
SEC24D
SF1
TBK1
USP9X
DAPL1
GABPB2
KLF1
MAGEH1
SNAPC3
BRD2

SLC39A10
AGMO
APOL3
ARL8A
EOGT
FEM1C
FITM1
NAALAD2
NDUFC1
RAB5B
RCBTB1
AKIRIN1
CHRNB1
COPZ2
LCP2
MBOAT7
NECTIN1
NMI
POLR1H
RAB11B
RSU1
SPOCK2
STMN3
SUMO2
VWA1
AP1B1
ARF6
CASZ1
CYP2C23
GPR146
LSM4
MECR
PXYLP1
S100A14
ANK1
ARAP2
DPEP1
FRMD4B
FUCA1
GALNT3
HSPB6
MMUT
ONECUT1
PREX1
RAB1A
REST
SLC6A13
SLFN5
SOBP
SUOX
SYVN1

TRAF4
ACTR3
CCT7
PLS1
PSMB6
FOLR1
SLC25A25
NSDHL
SCP2
OBP2A
PIGH
GNGT2
PTPN9
ZNRF2
IER2
ALDH5A1
CAVIN2
PSEN1
ATP6V1B2
DLL1
ITGA2B
RGS3
COX7C
CRTC2
CUL3
DERL3
EHBP1
FCHO2
GFRA2
HYI
ILDR2
ITGB1BP1
LPCAT4
MBNL1
MTRR
NCALD
PAG1
PKIG
POLR3B
PPP1R2
PRSS2
RLIM
RNASET2
RPIA
RPL30
SCN7A
SLC12A5
TBC1D1
TIAM1
TLCD2
TRIM59

UFD1
ZC2HC1A
AGXT2
DMTN
NCK2
NUTF2
REEP3
SALL4
SGTB
SIRT5
SLC17A8
USP3
SHMT1
EPRS1
ETNK1
ITGB8
PSPH
CYP1A
CYP3A5
AGR2
AUH
ERCC6L
GLIPR2
IPO7
MEIS2
MMP15
MUC2
PFAS
SAMD4A
SLC44A1
TUBG1
ARHGEF26
ATP5ME
CDK9
CDS1
CYB5R1
IKBIP
MFHAS1
MPG
NADK2
NUP155
SBK1
SGPL1
SLC12A6
TAOK3
YRDC
ACTA1
ACOT5
ANKRD33B
BCAP29
FAM13B

FKBP3
NDUFA8
PREB
PRODH2
SBDS
SNED1
THBS3
ABCB10
AP3M2
ATF6B
CBR4
CCNO
DBNDD2
DPY30
EFR3B
FIG4
GALNT11
KMT5A
MRPS28
MYH1
OPN3
RABAC1
RP2
SCYL1
SLC35E3
SLC35F5
TMEM177
TSPYL2
UTP3
BTBD7
CDKL2
NME6
SETDB2
SPIRE2
TUBGCP2
YME1L1
ZBTB34
AK1
CTNND1
KRT10
NTF3
CDH3
ATOX1
DNM2
FRAS1
MAPK6
MDM4
PPP1R14B
RPN1
ACOT13
ARHGAP22

CLIP4
CLK4
CYP2U1
ENAH
FGF10
JCHAIN
KPNA3
NDUFV3
NPTN
PHKB
PMS1
SMIM3
USP36
VGLL4
CXCL11
DDX3X
IL16
MDH2
FTL
CAPN3
CEP85
DENND3
GALNT7
HUNK
NDUFA9
NELFE
NUS1
PCBP4
RCOR1
SAMM50
SLC35B1
SPR
ST6GALNAC2
GUCY1B1
UCP3
ADGRA3
ANAPC5
ANKRD17
CES2E
HOXB3
HPS5
MOSPD2
MTFP1
PITRM1
RNF26
TMX4
TUBE1
ITGB5
SHMT2
ARL6IP1
CD200

HDGF
NOP2
NUMA1
TTPA
ALKBH7
DOCK3
DYNLT1
DZIP1
FOXJ3
LAS1L
PITX3
POLR2G
PTPRT
QTRT2
RFX2
SLAMF9
SLC66A1
TRAPPC1
PSMA5
CXCR6
CYP2C40
RNF186
TBP
ACIN1
AP1G1
CCNH
CTDSPL
FGFBP1
GMPR
HEBP2
NAGLU
PIAS1
STAT6
CYP2B3
FAM131C
ARID5B
LFNG
BDKRB2
C8G
CUL1
MXRA7
NRAP
OBSCN
P3H2
PGAP2
RDH5
SERPINA10
STAC3
WNT3
BCOR
CLSPN

KCNB1
PRKAB2
ENG
AKAP6
CIZ1
CTLA2A
CYP4A2
GTF2A1
MIA
PCMTD2
PORCN
PRPF3
REEP4
UBE2D2
VOPP1
GAL
IFNB1
CD70
CLMP
DAG1
GZMB
HEBP1
LARP1
MKLN1
PPP1R13L
SLC2A9
SLC38A5
TTC7B
ASH2L
CD200R1
LEFTY1
MAPKAP1
MRPL57
NHLRC3
SERPINA11
SHROOM1
SHROOM4
COL11A1
CRELD2
DYNC1H1
EPHA4
DAXX
DDAH2
FEZ1
GRPEL1
KTN1
RIOK3
RPL27
SLC41A2
STRA6
ZFR

ATAD3A
CEBPZ
EYA2
MARK2
MTFR1
NMRK1
OSTC
SNRPD3
THPO
TMEM120A
TNK2
TRERF1
HDAC2
CTDP1
HAPLN2
STK11IP
CACNA1A
RASSF4
CARD10
CGN
GPR137B
GTF2B
HECTD1
KAT6B
PKP4
PPID
TRAM1
TYK2
ARF5
ASB13
CUTC
DOK7
EID1
LONP2
NDUFS6
RBM17
SMC5
SUFU
TBCE
UBR3
ZFYVE26
SYNE2
TXNRD2
CCR5
ATP5F1E
ERBB4
MPRIP
PNISR
ABHD12
ALYREF
ANXA13

ASB2
DENND2D
FCHSD2
IPO9
KLC1
KRT13
LIMD2
LTC4S
NIBAN2
NRN1
PRPF40A
SCAF11
SLC16A12
USP13
WDFY3
ANAPC11
BHMT2
FBXL3
GBA2
HPS3
MOB3A
NUDT14
PARN
RT1-S3
WDR31
ATP7A
AHSA1
CARS1
GOSR2
NACC2
NAT1
RAB6A
RBM5
TMEM171
UROD
ATP2B2
CDON
FERMT1
GYS1
PADI2
PHLDB2
PPIB
RPS27A
ATG2B
CAB39
CHCHD7
CITED1
GPAT4
HLX
P2RX2
SH3BGRL2

SNTA1
TSR1
CDK20
GRTP1
LYRM1
SDR9C7
SENP5
ATG10
CCDC34
FGFRL1
GABRA4
RMI2
SCRN1
SNN
VPS13D
ZC3H13
PTGER4
ATF7IP
CSNK1E
ETF1
IRF4
MAP7D1
MOXD1
SEC62
SELENOW
BMP8B
CC2D1B
LRRC58
MAD2L2
MRPL47
MSI1
NEK1
NUDCD2
PCID2
PIGQ
PLA2G2D
ZNF608
NAA16
FBLIM1
PICALM
RPL27A
LRRK2
SERPINB9
CSNK2A1
SORBS1
AFM
ARHGAP20
CGRRF1
CLCN6
COMMD10
LDLRAD3

MIR222
NAP1L4
TIE1
TM7SF3
USP53
ABHD3
ADIPOR1
ARHGEF19
ATP6V1H
CBFB
MAPK8IP3
NDST1
NDUFA3
NMU
NPNT
PDHX
PELO
RBCK1
SBNO1
TRIM22
USP1
CYP4F15
DDX6
HUWE1
PDK3
POU2F1
RFC2
TMCC3
HOXD3
JKAMP
KCNF1
MED4
PEX11G
CA9
CAMKK2
EIF3B
FNBP1
FTCD
H2AZ1
HIBADH
MTA1
MYLIP
PRICKLE1
RPS5
RYR1
SERPINB8
TRIM25
APOL6
CPNE7
EMC6
FPR2

IFT172
PHF2
UCK1
ZFP62
ARRB1
CHI3L1
FAIM
FCGR3A
GRAMD1A
MAP4K5
MYRIP
NAAA
NIPAL2
NUDT12
PPP1R12A
RRN3
SDSL
UBL3
HNRNPU
C4BPA
CHD7
CHTF18
COL5A3
EML2
FRY
GCDH
GMPPB
GPRC5C
GSE1
HAP1
HLTF
LMO2
LRP10
MGAT2
MND1
NDUFV2
OAS3
PCDH7
PRXL2B
RAB27B
RPL10
RSRC2
SAA2
SEMA5A
SMARCC1
SNTB1
STRBP
SYBU
TBC1D8
TFB1M
IRAK2

ATG4A
CYP2D9
ISY1
KLHL12
MARCKSL1
NUDT18
PNMA2
SLC16A2
VIT
AFF4
HDAC9
HLF
IKBKE
KCNK1
MECOM
RAB3B
USP5
ABCB7
ARL6IP5
ARSJ
BACE2
CAPRIN2
DDT
DNAJA2
DPH6
GRB7
HFE
ING2
IPMK
LCMT1
PGM2
SAMD8
SPATA2L
SPIB
TOPBP1
TOR3A
WDR19
XRCC2
ADAMTSL4
CMKLR1
EFEMP2
EML1
EPHB4
FAM20A
FAR1
GTF2H1
MXRA8
OSR1
SCIN
SLC15A3
YPEL1

YTHDC1
ADGRD1
BAG6
BEST1
BLK
DPF3
HPS1
LARP4B
LZTFL1
MYOM2
PLEKHH1
RUFY3
SUSD4
SYTL1
TCEA1
ZBED5
ERO1A
CHAT
RXRB
SLC4A1
SLC4A7
AOC1
C5AR1
DYSF
SLC22A23
STBD1
TFDP2
TNIP1
CDC42EP3
DSG2
FGD4
HACD3
LSR
MYH14
PRRC2C
EDN2
FXYD2
GOLGB1
INPP5A
LYST
MOGS
MXD3
NME7
NXN
SIRT4
SLC35F2
SPHK2
THOC2
TMOD2
TRIM6
VAMP1

ABTB3
AMOTL1
BIVM
CEP192
CYP3A9
EDA2R
FNDC3A
GSAP
KCTD20
KDM2A
MYCBP
NEDD4
PCF11
PCNT
SEC22B
SFXN4
SMARCA1
TBCC
TMC5
TMEM11
TMEM147
TRAPPC4
TRIM44
TTC17
CABYR
CLEC4F
CSPG5
DCLK3
KLHL4
LIAS
MAML1
P2RY12
RBM10
SMNDC1
B4GALT1
CLIP1
ATP5F1C
CD93
CGNL1
DCBLD2
GNAT1
LURAP1L
PSD3
PSME3
TNFSF14
CD69
CXCL13
SLC9A1
TOP2B
AKTIP
BICC1

BNC2
BUD31
CXCR1
DYRK1A
ESPN
HAUS4
HJV
JAM3
JARID2
NDUFV1
PCDH9
RASGRF1
TMX1
GRIA3
KCNMA1
AOX3
CYFIP1
EIF3M
MGAM
NUP88
PBX2
PIAS3
PSMD5
RFX4
SBF2
SMYD2
TMC7
HOXC8
DOK1
LRFN3
MMP17
OSBPL11
RASSF7
SMYD4
ZFAND6
CLEC4A
CYP2B13
GPATCH4
KDM1A
KEG1
PSMC1
PSME1
SRPRA
CRAMP1
INVS
KLHL25
PTPDC1
TMEM267
ZFP503
LAMB1
CENPN

COL4A6
DEPDC7
DOCK11
ELL2
ERC1
GINS3
HIF3A
LHPP
LLGL2
MSLN
NDUFA1
PARM1
PDCD6IP
PPP1R14A
PRAG1
PSMA3
PSMB2
SLC7A6
SUN2
TOB2
ARPC4
LYPLAL1
MANSC1
MRPL38
MYH3
PRMT7
RIN3
SECISBP2L
SURF1
TBX15
TERF1
GASK1B
GTPBP2
LIMK1
MATR3
NCKAP1
PIK3IP1
RPS8
SLC26A2
SLC6A4
MUC5AC
ACOT8
CD79A
DHRS7B
EIF2B2
ELL
GGTA1
H1F2
HDAC11
KCTD9
LEO1

MAGT1
MIR29C
RALY
RSRC1
SPPL2A
TERF2IP
TMEM150A
TMEM41B
TTC14
ALDH3B1
GALK1
CELF1
ESYT1
GRAMD1B
GYPC
HLCS
LIG4
PGP
PI4K2B
RARS1
SLC14A1
SLC30A10
TUBB4A
UBAP2L
ZRANB2
NR5A1
SHANK2
CASKIN1
COL24A1
IL20RA
NARS2
USP20
CYP3A44
FOXS1
NVL
SOWAHB
DLST
EEA1
ERCC2
FAM210B
JAM2
MUP2
PTGFR
RCC2
RGL1
SPAG9
STMN2
ZFP52
AGFG1
CALCRL
DDX23

DEGS1
FAM222A
GRIA4
HIPK3
LRP12
NCK1
NEIL1
NOC2L
NUDT5
RGS20
RHOD
RND2
SFXN5
SNRPE
TMEM106A
ACTC1
RPS7
IDO1
B3GNT2
BCL2L13
CHST1
DDIAS
LOXL4
MN1
NPPC
PLBD1
RHBG
SEPTIN11
SLC12A3
SLC25A24
TNFRSF14
ACAP2
AGTRAP
BNC1
CES1G
GCN1
GDNF
GIPC1
MICB
MRPL46
NELFA
PPIL4
TRIP10
CAMK1D
CSNK2A2
CYP51A1
PDE1A
TREM2
CALU
GCHFR
PLN

SEL1L
SST
BTN3A2
DHX35
H3C15
MED27
MMRN2
NSMCE2
POLDIP2
PPP1R11
PSENEN
RNF130
SEC22A
THG1L
ZDHHC9
ZYG11B
ARHGEF28
CAV3
CHIL3
CKLF
DNAJC1
EHBP1L1
FAM89A
HOGA1
KLHL13
LYNX1
MAX
MLC1
MOCS1
MPC2
NDUFA13
PCLO
RDH16
RLF
SLC45A4
UGGT1
ASCC3
BACH2
CLIP2
CSF3R
EGFL7
PHF20L1
SLC22A12
SLC31A2
INTS6L
ZDHHC16
BOP1
CCR6
DAPK3
FZD6
RABGGTB

SLC12A7
CPEB4
FARP1
HSPA6
SARS1
PTGER2
CES1E
CSGALNACT2
NDFIP1
NDUFA11
NHSL3
ONECUT2
PACC1
PRR7
PRRX2
SGCB
TDRD3
TP53I11
TWNK
VEZF1
ZFP91
ZFYVE1
HIGD2A
M1AP
THEM5
ADAMTS12
AHCTF1
AOPEP
ARHGEF16
CSPP1
FAM117B
NKD2
OLFML1
OSGIN2
PTPA
SCFD2
SMURF1
SYTL4
UTP15
ARHGAP45
BPHL
CPAP
GMDS
ITPRIP
NTS
RBX1
SOX5
SPAG1
TTC39B
UQCRQ
ACTR10

ELP5
LCMT2
MRE11A
MTREX
SHLD2
VARS2
CEL
KLKB1
RARRES2
SPC24
TOMM40
TRPV2
VAMP8
SIK1
ACSM1
DAZAP2
HSD17B13
KIF4
MRPS33
PDCD10
RAB15
RAB25
SFRP5
STAM
TDRP
THUMPD1
TMCO1
TSPAN14
GSTZ1
CELA2A
GPSM3
HOXD10
SUPT6H
AP2A1
ARHGAP1
ARL3
CDK19
CEP152
CHST14
CHST15
ERCC3
GEMIN6
HDAC8
IL20RB
MSRB2
NEURL1B
PARP2
PRKX
RAB5C
RFLNB
RNASEH2B

RPL31
SERPINA5
TMEM106C
TRIM13
USP47
VGF
WDFY1
ZMYM2
ABCA2
ACBD3
ANKRD10
AQP5
ARID5A
ATP5F1D
CASP8AP2
CENPI
CNTRL
COX7A2
HNRNPM
KANK4
MPC1
PLA2G4C
PLEKHA2
ST14
TMEM33
TNFA
ITPR2
L1CAM
MTRF1L
CD58
CR1L
E2F6
MAST3
NKIRAS1
TMOD4
TMPRSS6
ZFAND2B
ERCC6
F11R
KCNQ1
PEG10
PEPD
CYP2S1
DPT
PON3
PPM1B
SERPINI1
ARHGEF9
CLIC6
CMTM8
DYNC2I2

FAM241A
LHX1
LSAMP
NDRG3
NDUFB9
SLC25A32
SLMAP
TMEM107
ZFPM2
ACP2
BZW2
GDPD1
GMPS
TLE4
TSPAN12
ADTRP
AFAP1L2
CD79B
CNTNAP1
DCAF11
F2RL2
NIT2
NOL6
NPHP4
POLK
RHOJ
RNF19A
SEC61G
SLC25A11
SMURF2
TBC1D5
TRAK1
FBXO34
KRT14
PRKAR1A
SULT2A2
ZNHIT3
ATP2A3
MXD4
PECR
TRPM7
VSNL1
WDHD1
ACP1
ADPRHL1
APOOL
ARPC2
AZIN2
BST2
DHX38
EIF1B

ELL3
FLRT2
GRHPR
KDM5B
LGALS2
MAG
MRPL2
MTARC1
NRG4
PEX11B
PIGT
PIP4K2A
PPM1M
PRUNE2
RNF170
TECR
TM4SF19
VBP1
WNT5B
ABCB9
ARMC9
CHN1
DGCR8
GAS2L3
GEMIN5
HCLS1
KDM4B
NOL8
RNF128
SNRPA1
SPNS2
TRIM47
TTC39A
VRK1
CDADC1
CTNNBIP1
CYTH1
FER
FRK
GALK2
HBS1L
HOMER3
KLHL7
LRRC47
NBEAL1
PALD1
POLR1E
SAP30BP
SURF6
TMED7
TPPP

AS3MT
LIPA
LONP1
ACSM2
CLCN3
HNRNPL
LMNB2
PPA1
SGO1
TTN
VAMP2
ALS2
CD22
DSCC1
GALNT1
MIDN
MYL4
PLOD3
RERE
RPL9
SDCBP2
UGT2B17
CACNA1G
FMR1
PARP14
CFAP36
CHSY3
PELI3
PLPPR2
POT1
UPRT
VSTM4
ACBD4
AIMP2
ALPK2
CD164
COX15
CPPED1
DMAC2L
IKZF2
KCNJ5
MKNK1
MYBPC3
NAB1
NDUFB10
PITX2
PPP1R14C
PRICKLE2
SGPP1
SH3BP4
SP5

TLR8
TMEFF2
APEX2
CCDC136
CFAP251
CHID1
CNTFR
DOK4
DYM
FBXO22
HYKK
LRRFIP2
MAP4K3
MARCHF2
NMD3
ORMDL3
P2RX1
P2RX5
P3H3
PDE6C
PMS2
POGLUT2
POLR1F
POMP
SCAND1
SHISA3
TBC1D17
TM9SF2
TMEM154
UGT1A5
ADARB1
FARSB
RBMX
PDE2A
ACTN2
DCAKD
GCKR
MACROH2A1
MAST4
MYOD1
RAB20
RPL37
SYN2
TMED5
AHCYL2
AMPD2
ANKH
APMAP
ATG4B
BTD
DMXL2

FA2H
FBXO3
INTS6
LRRN3
PCBD1
PLEKHO1
PURA
RALB
TCEA2
TFEC
MEF2D
NDUFB8
RPL22
SND1
B4GALT2
CALN1
CASQ2
DAPP1
DESI2
ESRP2
ITSN2
NSD1
SCUBE2
SGSM3
SH3D21
SIL1
TIAM2
TIMM23
TUBGCP3
ITGAL
AFG2A
COQ5
CWC27
CYTH2
DDA1
DNAAF5
FAM133B
GOLT1A
MESP1
MRPS21
NAT8F5
NEDD8
OBI1
PRPF18
PRUNE1
PSMG2
PUS10
RALGAPB
RNF138
TFF2
TMEM120B

TMIE
TRIM9
UBXN6
VNN3
WIPI2
ADAR
AP2M1
AZGP1
CHD3
CYP2J2
GAS2
MTCH2
SMAD9
AKT1S1
COX5A
EHD1
IL10RA
SERPINA3N
WDR1
CDK12
CHCHD6
HABP2
HINT3
INPP1
MCFD2
NDUFB3
PTPRR
RREB1
SFMBT2
SIDT2
TRMT61A
VAMP5
ARHGAP29
ARHGEF7
ATP5MF
ATRNL1
CIP2A
DOP1B
EPHA5
IK
KCNS3
MICU1
NDUFB7
PARP3
PIGP
POLR2H
PTPRS
RBFOX3
RPL14
THOP1
THSD7A

H4C12
HOXA1
ART4
ATP13A1
CHST8
CLDN23
CYTH3
EGLN1
EIF1A
ELAPOR1
EMC8
FAM20B
FCGR1A
KLHL9
MPPE1
NEU1
PHKG2
POLE4
PRAF2
PREX2
SDHAF3
TBPL1
TTC12
TTLL3
URB1
GH1
TAC1
B3GNT9
SLC17A4
ACTR2
AIF1L
BANP
CCDC88A
CENPH
COX7A1
FHIP2A
H2-K1
IDH3G
KLK1
KRTCAP3
MMP23
ND4
NYNRIN
OCIAD2
PIEZO2
PLEKHG5
PPM1H
SEMA4A
SERINC5
SLC27A3
SRD5A3

TAF5L
TUBGCP5
BAG1
COBLL1
OSTM1
SPRED2
LIMCH1
LTA
NECTIN2
TP53BP1
CD19
CLASP2
EXOC6
FANCB
FUT1
FYCO1
GABARAPL2
GLIS2
MAP3K3
MRPL4
MYL12B
NCOA5
PAFAH1B2
SNAP29
SPIN1
TRMT10A
VGLL3
WNT2B
ADM2
CCR3
DDOST
EXOSC5
GRK6
MAPRE2
POLR3E
SAMD9
SDAD1
SF3A2
SNRPG
TTYH2
USP25
ARC
CHRM3
ARMCX2
BTRC
CFDP1
HDDC3
MYH2
NCAM2
PUS3
RGS11

SENP2
SGSM2
TRIM38
UTP11
AKAP8L
CPM
EPSTI1
HEXIM1
ICAM2
MIR155
NSFL1C
PHB2
PPP1R9A
RPS11
TMEM37
AMY1
FAM120B
MAN1A
OXNAD1
RANGRF
RING1
SLC41A1
SUPT3H
CARM1
CCDC18
CHP1
CKMT2
DHRS1
DPM3
GLCE
JPH3
KCNG1
KRT6A
NAPSA
NSD3
PALS2
PRPF39
PTK7
RAB21
SOX7
SWAP70
TSHZ2
UNC13B
MIR16-2
MUP5
NAT8F1
TGS1
AQP8
COL4A3
DNTTIP2
EMG1

EMX2
FN3K
LSM2
PCMTD1
POLR1C
PRKD2
PSMD6
R3HDM2
SH3RF1
TSPAN7
RANGAP1
AGPAT3
EML4
KANK2
LMO4
MFAP5
MIR122
P2RY1
PRRX1
RPN2
SCG5
SEPTIN4
SRSF6
AMHR2
BICD2
CENPP
DLX4
GATA5
GREB1L
HELQ
KCTD3
MAGI3
MAK16
NOL11
NOP14
PHF8
PWP2
RNF44
RNF7
SHOX2
SNX14
TMLHE
TRPV1
TTC23
UBALD2
ZNF503
PRKAR2B
PTAFR
ATG14
CCDC68
FAM110B

GIGYF2
GNG5
LAMTOR2
LAMTOR3
LMOD1
NUP160
PFDN2
PLEKHH2
PRMT2
PXK
SPIRE1
TTC27
CHD2
MYO10
UGT1A7
BBX
CBX3
CDC42BPB
CYP2C70
DESI1
DOCK4
EXT1
GMFB
HARS2
KIF5C
MORF4L1
PHACTR2
PPP2R5E
RGS18
RHOT1
S1PR2
SDR39U1
SEPTIN8
SETMAR
SLC12A8
SLC25A15
SSPN
ST8SIA6
TIMP4
TMEM138
TMEM192
UPP2
YES1
ZFP367
ZFTRAF1
KNSTRN
CAAP1
CYP2C7
MPND
PARL
TYW3

NPY1R
RPS6KA5
ACP6
AMFR
C1QTNF5
CBARP
CLDN15
LPAR2
MRPL9
MUC13
MYOM3
NXT1
PHF5A
PLCL1
PTTG1IP
RBM34
UNC119
FAM111A
FRMD4A
LHFPL4
PPY
PSME4
BLZF1
DDX56
EXOSC4
GTF2H5
MAP4K2
PDE8A
PDSS1
RHOF
SERTAD4
TFAP4
WWP1
ADAM22
CIART
CLIC3
FANCG
FMOD
KCNAB2
OSR2
PPP3CB
RNF145
SEMA3F
SLC25A36
SP100
BMPR2
GHRL
POLB
SIRT2
UBB
CCDC127

COX4I2
FBXO41
FBXO8
IQCK
OBP3
RHBDL1
STYX
UMOD
UNC50
ZBTB8OS
NOX1
MSI2
PSMD8
SF3B1
SUV39H1
GNAI1
ACTR1A
ARHGAP30
ATP6V1F
B4GAT1
CHPF
DENND4C
ENHO
ETFRF1
HSPB9
ING4
KLHDC8B
MIR30A
MRPL16
PTCH2
RABGGTA
ROMO1
SAA4
SNIP1
ST6GALNAC3
UBIAD1
AADAC
CELSR2
EIF2AK4
HSD17B8
SELENOS
SNRNP70
TNFRSF10D
TOX3
ARF3
CTDSP2
DNAJC21
DVL2
GPD1L
KLC4
SART3

TRIM27
UTP4
ANK
FBXW9
FUT7
GPANK1
UBE2J2
G3BP1
ASAP1
CAP1
EPS8L2
ETV1
SLC46A1
ADCY2
ASB14
DEDD2
EP400
FXYD1
REEP1
SNRPB
TICAM1
ADCK2
ANGPTL1
CACNA2D4
CLEC4A3
CYP21A1
HSD3B3
IFNAR1
LRSAM1
MGA
NICN1
SIDT1
SLC4A3
STAP1
HMOX2
ALMS1
ARHGEF1
ATP10A
C1QTNF4
CLPP
CMTM7
CNNM2
DHX36
F11
FHL3
FKBP8
FYB1
JPH1
MED13
NHLRC2
PRPSAP1

RFK
RNF152
TLK2
TMEM117
TXNDC16
WNK4
AP2A2
ATE1
B3GALT1
CRCP
DCAF13
DCAF7
DEF8
GNPTAB
HIKESHI
INKA2
MRPL3
NDN
NUB1
P2RY14
RGS7
RORB
SCRN2
TBCEL
TLE5
TM2D1
TMEM156
TMEM9
TSPAN6
UBE2Q2
FGF9
TRH
SNORD22
EFNB1
SLC27A4
ADORA1
ACTN4
DNAJB6
GABBR2
GFPT1
IRF2BP2
APBB2
CD28
PPAN
PTPN14
RGS12
RPS28
CUZD1
DNAJC17
FBXO42
HSD3B6

LTB4R2
MYO18B
TSTD2
AMPH
AP3M1
ARHGEF12
CRLS1
IRF2BPL
ITGA9
KCTD1
PDS5B
SLC6A14
SPI1
TMEM45A
TRRAP
WTAP
GALNT2
RCN3
SUMO1
AIM2
DOK2
EFCAB7
FAM72A
FASTKD1
GET1
IPP
KLRK1
LIN54
MIR27A
MXRA5
NEIL2
NEMP1
SPATA2
SPG21
TRABD
TUBD1
VRK3
ACSM5
AMMECR1
AMT
CEP170B
CNMD
CTNNBL1
DDX27
GLOD4
HNRNPUL2
HSPA14
IL21R
NKX2-1
OSBPL10
PIP4P2

PIP5K1B
PLEKHF2
PUM1
REG3G
ROPN1L
RYK
SH2D3C
SULT5A1
SUSD1
TMEM144
VTI1A
FDX1
CDCP1
CELSR1
CLSTN1
CTDSP1
DOCK5
HS3ST1
IRAK3
KIF5B
LDLRAD4
LHX2
MIS18BP1
NRXN3
PLXND1
PRR5L
PSME2
SIVA1
SLC3A1
SVEP1
TUFT1
CYP2B15
HIVEP1
NCF4
PDLIM2
AQP11
ASAP2
C1GALT1C1
CHIC2
CREB3L1
DIAPH2
DTNB
G3BP2
GBA1
OSBP2
PBRM1
PIN1
RAB40B
SCAMP5
SETD5
SETX

SOWAHC
TANC1
TMEM123
UBE2O
ALOX15
CRIP1
KIF1B
NFATC2
AAMP
C2CD3
CCDC3
CES1F
COXFA4
CRTC3
FLACC1
GMPR2
GNB5
IL18RAP
KLHL2
LSM10
MLF2
NDUFB4
OTULINL
RASSF6
SFI1
SLC35D2
SUGT1
TMC4
TMEM42
TMEM44
VPS33A
APOO
CLEC3B
DUS2
GIMAP6
HPGDS
HTRA2
ITM2B
JADE2
NDE1
NUP50
PLXNA1
PTBP3
RNH1
SGSM1
STIL
TAB3
TMCO6
TMEM69
TPBG
TPCN1

ZBTB46
DOCK8
UCK2
DICER1
MAFK
C4A
DCTD
ESAM
FBXL4
HBA-A1
IFT80
IGDCC4
IL27RA
KDM5A
NPTX2
OTUD7B
POLI
PPP2R2C
PUM3
RAP2B
RMDN2
SCN3B
SLC16A11
SNRNP200
TIMM13
DAB1
DPP7
NDUFA2
PIK3R5
POLR3G
PPBP
SLC5A6
TAOK1
LOXL1
ADH5
ARHGAP21
BANK1
BDKRB1
CD99
CX3CR1
GXYLT2
HMGB3
HNRNPLL
HSPG2
IFT57
KLHDC7A
LINGO1
LMO1
NAV3
PFN2
PGGT1B

RMND5A
SRSF3
TBC1D24
TUT4
USP12
ZBTB7B
MFAP3
CALCA
DDX31
DTD1
FAM219A
FBF1
GGA3
GPR153
KCTD17
LZTS3
MRPL30
OSGEPL1
PHF12
SEMA6C
SLC35B4
TCTN1
TDH
TRIM11
TRMT2A
TUBA1B
APBA3
MBL1
MEP1A
THAP4
ZFP207
ATP2C1
BHLHA15
CLN3
COL9A2
DMKN
DNAJC19
EAF1
FIGN
FMN1
H2BC12
MRPL23
NME3
PAK3
PARP8
RIT1
SASH1
PIK3CG
UBA1
COX7B
DNAJB2

GCNT1
GNG2
KYAT1
MAP7
NPTX1
PIK3C2B
RBM47
RPL5
SETBP1
SLC25A12
STON2
TPPP3
CERS4
CNOT1
CSTF2
CYB5R2
EIF2AK1
GAS2L1
GUK1
HS6ST1
MRPL45
PACS1
PRMT6
QSER1
RNF149
TAF9B
TSPAN31
TUB
TUG1
UIMC1
WBP1
ZNF395
INCA1
PXN
GAD1
CALCOCO1
CAND1
DERA
EIF4E3
FLVCR2
GOLT1B
LARP7
NDUFB5
PDLIM4
PGAP1
RAB38
SNX29
SPOCK1
TMEM39A
CDC7
POLR2A

ANKRD6
BRINP1
C1D
CAPN15
CCDC90B
CCL21
CDC123
COG8
DDX59
GDF3
GPR3
GTF2F2
H2-EB1
LILRB3
METTL5
MORC4
PAIP2B
PWP1
RBBP5
RFLNA
SLC2A14
SPNS1
SPSB3
TBCA
TMEM254
TMEM59
ZDHHC20
ZNF703
CHRDL1
EOMES
EYA1
NHP2
PNPLA7
SAFB
SNRPA
TNPO1
ZMAT5
B3GALNT2
CYP2C12
GPR27
HC
HSPB3
INTS1
MED18
MYOZ1
NOS2A
SLC16A8
TNFAIP8L2
ZNF385B
DEK
NAV2

AGA
BCL2L14
BRI3
BTAF1
CEP78
CKMT1
CORIN
GTF3C2
HCFC1R1
HDAC10
HSD17B14
MAZ
PLD4
RHOV
SFMBT1
SMG1
STN1
SUSD3
TBX1
TMED4
TOR1B
TRIAP1
VANGL2
DNER
PDE4A
ABCF2
ELMO1
IL17RD
ITPKB
KCNE3
NAT10
NCAPD3
NPR1
PMM2
RPL35A
STUB1
UBE2K
BPGM
C7
DCX
HS3ST3B1
NRXN2
RPL26
SEC23A
SPIN2B
ATXN1
CYP2D6
ABI3
ANKRD46
ARMC1
ASB9

BARX2
CD180
CFAP20
EIF3K
IKZF4
KBTBD2
LONRF2
MOSPD3
NIPA1
NOL10
PEX16
PTPN23
RAB11FIP2
RAD1
SCTR
SSH2
USP6NL
CAMK2B
AGMAT
ATP6AP1
AVEN
CENPL
CEP70
G6PDX
GKAP1
GOLGA4
GPR68
GRK3
JAZF1
KAT5
PRRC2B
RELL1
RIN1
SEMA6A
SRRT
TSEN2
ZBTB38
ANGPTL8
ANKS6
DYNLT2B
METTL27
PCNX3
PDE6D
RCCD1
RTL8A
SSH3
ZGRF1
ACSS3
CAMTA1
CAPN6
CKAP5

COPB1
CRLF1
ITPK1
RECQL
TAB2
TMCC1
ANKRD13C-DT
SCNM1
SRSF2
ZFP385A
FAM76A
LRRC24
LY6C1
SLC25A18
UPK3A
MAFG
SLC18A2
NFIC
RPL13A
ADRB1
GNAS
AIP
CADM4
CHRD
ECE2
FEM1B
FKBP7
GMIP
GPR157
MARCHF5
METTL16
MIPOL1
MRPS26
NUFIP1
PRELID2
RIOK1
RIOX1
TMBIM4
TMEM25
ZFP64
COX6B1
DDX39B
ERP44
INA
PAX3
PGAM2
RASGRP1
SLC4A2
ANKRD28
APOBEC1
ATRN

BCKDK
CPSF6
CPVL
CTBP1
CXCL6
DDI2
DENND4A
DHX15
DHX30
DUSP11
DUSP9
EIF1AX
FAM3C
FNDC1
FOXF1
FRMD8
HECA
HIRIP3
IDH3B
LANCL1
LNX1
LRR1
LTV1
MASP2
MRPL19
NANOS1
OPCML
PLSCR4
PTPN7
SASH3
SLC39A5
TEC
TEX2
VPS37B
WNT10A
YKT6
YOD1
AKR7A2
CYP2C
DDX51
EXOG
FUZ
GALNT9
GPN2
RNF185
RNF187
RPUSD3
TEX9
TMEM9B
TOR2A
DMD

SH2B3
TUFM
ATP5MG
BCL9
NANS
PCDH18
PRCP
RNMT
SIN3A
SPCS2
TDP1
UACA
UPF1
ANK2
APPBP2
ARL2BP
ATIC
BAZ2A
CCDC88B
CEP95
CRTAC1
CTR9
DUSP26
GET3
LPCAT1
LRRC15
MIF4GD
MRPS18A
NT5C
RBM15B
SERAC1
SSH1
STK25
TMEM164
TMEM71
VPS8
ZP3
ARMCX5
CYP2T1
KRT86
ZFP148
ZNF362
AFMID
ARVCF
CIB2
CLN6
CMSS1
DDX11
DGKZ
EMC1
FKBP9

IMMP2L
KCNIP3
KCNQ3
KDSR
MAFA
MAN2A2
MAS1
NFASC
NLGN3
PAPLN
PLPP5
PWWP3A
RETNLA
SCOC
SH3GL3
SNRNP25
STK40
SYMPK
UBR5
VCPIP1
XPO4
TPO
SANBR
AMOTL2
HEG1
LUC7L3
SEPTIN9
SRSF10
SULT2B1
WNK1
AKAP13
NOP58
UGT1A8
ABHD11
BBS7
BCL2L12
CDC42EP4
CDK5RAP3
CLCN5
CYP2C6
GDAP2
HELB
LRRC51
MYBPC2
NELFCD
NHERF2
PARVA
PASK
PCSK1
POU3F1
RNF34

RPL36A
SENP3
TNFRSF10C
TRPC4AP
TSPAN15
TTC8
VPS37D
WDR37
XKR8
ZDHHC3
ANKRD44
AP1S3
AP2S1
CAMK1
CDIP1
CEP128
DGLUCY
DOCK6
FAM168A
FAM171B
IGSF11
MRPL20
NOVA1
PANK3
RALGAPA1
RIC8A
SLC17A2
SMAGP
TBL3
TRAPPC6A
BAG4
CCNJL
CGAS
CRYZL1
DEGS2
EXTL3
HECTD2
HSBP1
NDUFB1
PAFAH2
PEX5
QTRT1
RHBDD1
RUFY2
SEC11A
SNAPC1
SNTB2
TSC22D4
TXLNG
ZFP354A
ADCY9

CCS
DDX60
DPYSL4
RPL36
TNNT2
YPEL2
ZMYM3
DHRS11
PIK3C3
RBM39
B4GALT3
HEMGN
ICE1
KBTBD12
MCEMP1
ODAD3
SLC28A3
SLC7A14
SPICE1
UGT3A1
ZNF143
ADRA2C
CCL24
DDX18
DPP3
GJC1
H2AZ2
HIC1
IMMT
LPAR3
PITPNA
POLH
PPP2R3A
PUS7
TRPS1
ATRX
FOXP2
SPTBN2
KL
TNS3
ADORA3
BCL7A
CDKL5
CDX2
CLPTM1L
CLTA
DSN1
DTWD1
ERBIN
GABBR1
GNL2

KCNA2
MAN1A1
MTMR4
PYROXD2
RFX5
RUSC2
SEC24B
SESTD1
SKA2
TMEM163
ZBTB18
ZFYVE16
IFI27L1
RPUSD1
S100Z
SLC35E2B
CENATAC
CENPO
COLEC11
DHX57
DIMT1
ICAM3
KCTD6
KIF9
METTL18
MYLK2
NUDT13
OCRL
PLEKHG6
RADIL
SLC7A6OS
TCF15
VPS39
XAB2
ZKSCAN3
ABCG4
ARHGAP4
BRAP
CALHM2
CBLN1
CCDC126
CCP110
CLCN2
CRELD1
CSDC2
DPH2
HOXB6
IL22RA1
NOXO1
OAZ2
POLR2I

PRRG1
SLC2A10
SLC7A9
SPAST
TMEM208
TOMM40L
TRIM32
TSN
VPS33B
XRN1
ZNF552
CACNA1C
IL24
ACVR1
CPB2
CPZ
KYAT3
LTBP3
MYL12A
OMD
DNPH1
FOXC1
PSMD11
SERBP1
UBQLN1
SLC22A6
CRBN
CUBN
EIF3F
GBP6
IBSP
IRX1
MRPL50
NHSL1
PCP4
PHYHD1
PMPCA
PPIH
RPL41
SDHD
TRPM6
WDR45
ZBTB7A
AARD
CABLES2
CYP2C50
NLRP6
VPS9D1
ADAMTSL5
ADAT2
CHRNA2

CLCC1
CTDSPL2
CYTIP
HAUS1
HVCN1
HYLS1
LDB2
MAGEE1
MARCHF7
MNAT1
NUBP1
NXT2
PGGHG
PYGO2
RAP1B
RBM33
RMDN1
RSBN1
SPATA6
WASF1
TRPV4
ALKBH1
ARHGEF25
AUNIP
B3GNT3
CHODL
FUNDC1
GTF2H2
HSF2BP
KLHDC10
LNPK
MKX
MTX3
NOL7
PIGF
SLC6A17
SOCS7
TATDN2
TM2D3
ZBTB14
GNA13
MAN2A1
MOCOS
ATP9A
DENND5A
FUT4
GFM1
GPR176
LUC7L2
METTL1
MLLT10

PTPN22
RAB8A
RALGPS2
SAPCD2
SLC30A3
SLC30A7
TBRG4
TMCC2
TPST2
CENPC
ECI3
LDOC1
PRKN
CASK
COCH
GAS5
INHA
INPPL1
KCNJ2
MAP2K7
MNS1
PGLS
SNRK
CAR14
EFNA4
EID3
EPX
FAXDC2
IFT74
KDM5C
KIFAP3
MAP3K13
MBD6
NAPA
NOP53
PABIR2
PRR16
SEZ6L2
SIRT7
SLC15A4
SNAPIN
STXBP4
SYNJ2BP
THNSL1
WRN
ZBTB5
ZDHHC21
HPR
NICOL1
CMKLR2
CYP4F11

H4C11
HAMP2
HOXC9
HPF1
KLHL8
LRCH3
LY6C
MIR223
RPS19BP1
CHIC1
CNIH3
COX20
DCLRE1B
DOLK
DYNLRB1
ECEL1
EDARADD
EYA3
FOCAD
IFT46
LSM8
MAPKBP1
MPHOSPH8
PIGM
PMF1
RHNO1
RNF220
ST8SIA3
WDR35
ZDHHC7
GAD2
GNB1
ACOX3
ALPP
ARPP21
CD3E
CTF1
CUL4B
DDX54
ELF4
ERGIC1
ESRP1
L3MBTL3
MTARC2
NSMF
RAB23
RHBDD2
SBNO2
STRN3
SUMF1
TAF13

TET3
UNC13D
BAIAP2
HINT1
ADGRG6
ATP6V0C
CHD9
EMILIN1
FOXC2
HDAC7
KCND3
LAT2
MCM8
MLLT3
PCBP1
PKD2
SAR1B
SELPLG
STAG2
TBC1D15
TMTC1
TRIM14
DPYSL2
PVR
ABRACL
APOL9A
ATP11B
B4GALT7
CASD1
CEP290
CHMP2B
CMPK1
COX11
CPXM2
DACH1
FAP
GMPPA
HYCC1
LCT
MPHOSPH9
NTNG1
PKN1
POGLUT1
PPHLN1
PPP1R13B
RRAGA
SERPINA3M
SGCG
SNRPB2
SNX1
STAM2

TSSC4
C2
PLP1
RPL24
ANKMY1
BHLHB9
FOXN1
ATXN3
BCAP31
CCNL2
ERG
HNF1B
ISCA1
KAT14
KRCC1
LYPLA1
MTFR2
NRIP3
PITPNB
PLOD1
PXDC1
REC8
SENP6
SHANK3
SLC2A12
SPTLC3
SYNJ1
TBL1X
TDRD7
TFAP2C
UVRAG
VDAC3
ALOX12B
DLGAP2
HOXA11
LYZ1
NAA35
NAGPA
NUMBL
PTOV1
RBM7
RFXANK
TANGO2
TCP11L1
UBL7
ZNHIT2
CCL22
SLC6A3
APCDD1L
BICRA
BIRC7

CRNKL1
FADS6
MAD2L1BP
NATD1
NPB
ULBP2
HSD17B1
ALG3
CCDC25
CEP350
CLDN10
ERLIN2
GLT1D1
GPR35
H3C10
HOXB5
KLHDC8A
LRRN1
MARCHF3
MCEE
MEGF9
METTL8
MINDY3
MIS12
NRBF2
NUP54
PDCL3
PLXNA3
RBM18
RBM28
RXYLT1
SH3GLB2
TMEM51
WHAMM
XYLT2
YAF2
AFAP1
BAP1
GANAB
MRTO4
NIPBL
PPP2R1B
RGMA
SEMA3E
SEMA4D
JMJD6
PLCG2
FOXJ1
GAK
GALNT12
ICE2

JPT2
KCNJ12
NOD1
PJA2
RAB2A
RALGAPA2
RBFOX2
SEPTIN6
SLC26A1
TMEM19
TRANK1
TXNRD3
GNRH1
PDIA5
ADGRF1
ANKRD16
BRMS1
CCDC91
DCUN1D5
DHX16
FMNL3
GTF2H4
KLHL22
LLGL1
MARK4
MMGT1
ND4L
NGRN
PHLPP2
PLAAT4
POGLUT3
PSD4
RT1-DA
SETD3
SIX5
SMARCAL1
SNAPC4
STRADB
TBC1D7
TMPRSS4
TTC38
PLIN1
MT1X
ADCY1
CPLX2
GPR183
NUDC
PLEKHA6
RPS17
RRP9
SFXN3

STARD10
APOL7A
B3GNT8
HOXC5
ADAMDEC1
AKR1C6
BAIAP2L2
BOLA2
CYB561A3
DTX2
FOXD2
MORC2
REG4
SNHG32
VSIG10L
YIPF1
AFG3L2
AKIRIN2
ARPC5
CADPS
CAPN8
EIF3L
FAM78A
GAPDHS
GCSH
GHRH
GXYLT1
H4C16
HS6ST2
INPP5B
KCNT1
LARGE1
MORC3
NCAPH2
NUDT16
PLRG1
RAB34
RHOH
RTCA
SAMD11
ZFYVE28
ZW10
ALG2
AP2B1
C2CD2L
EXOC3
GLRA1
IL32
KDM2B
MFAP3L
NDUFA12

PDRG1
PFKFB2
PNLIP
RAB4A
SIRT6
SLC44A3
TM9SF3
DHX58
RPL18
PIK3R2
PTPRJ
STX1A
CDC23
CDC40
CHCHD1
DDX25
LCLAT1
MAEA
PGLYRP2
PIGW
RWDD3
SCAMP4
SDF2
STX17
SULT2A6
TNNI1
VPS52
VTI1B
GRIA1
ADAP2
AEBP1
AKNA
ATG16L1
DAP3
IFI44L
KCNAB1
OSER1
PALM2AKAP2
RAB10
RPTOR
SARAF
SPTB
STEAP2
STK17A
TENM4
TMEM50B
UBTF
ADH6
AHDC1
AKAP11
ARL15

CD320
CNNM3
COL9A3
HLA-DMB
NDUFB11
NT5DC3
NUP107
PBLD
PRTG
SDF4
SPTY2D1
TMEM119
TREM1
UBE2A
UBE2M
ZFP37
ZNF367
REN1
SUPT4H1
CCDC106
COL26A1
DNAAF3
PSD2
PTCHD4
SERPINA9
TADA3
TLCD5
TMEM184A
ASCL1
CSNK1D
EBI3
GLI3
MT1H
PDE7A
SORCS2
SRP9
WASL
ANKAR
CIPC
KANSL3
LRTM1
OAZ3
SNX19
ZDHHC19
H4C8
LAMA4
FGD3
ITM2A
JPH2
NDUFS7
RAB3IP

SCGB1A1
WNT2
ALKBH8
APOC4
CCKAR
CHERP
CRLF3
DLK2
FAM81A
FRAT1
FRRS1
FUBP3
GLB1L
MARVELD2
MED20
MIER3
POPDC1
RGS8
SMCO4
SPAG4
TEX261
TNFRSF18
UBL5
ZFAND4
CD2BP2
CRHR2
ELAVL3
HAVCR2
ISCU
KCTD10
LRP3
MUSTN1
PROCA1
PTS
RAB13
RFNG
RILPL2
SPP2
VWA8
XPO6
XYLB
GAP43
FAM98A
GDI2
KIFC3
PAX8
PCBP2
SCG2
STING1
WNT1
LAMP3

RBM13
AFF1
C1QTNF1
CHST3
CNBP
DLX5
EHF
HACD1
TEX264
TTC28
ZBTB44
BMP15
CST7
GRPR
HECW1
METTL15
PIGV
PKNOX1
RAB4B
TMEM203
TRIM45
TRP53INP2
BCR
CGB3
CHD4
CNTN1
COL16A1
FURIN
GYG1
IGTP
RASA3
TAF15
UCHL3
ADRM1
ARHGEF40
CDK10
ERGIC3
GABARAP
MBLAC2
MINPP1
MLX
MRPS22
RASSF8
RPS12
SCG3
TRDMT1
UBL4A
WBP2
CAPN10
CEP83
CYP4A3

DNAJC13
DNAJC8
DUS1L
EID2
IGSF8
KIF16B
MAP3K7CL
MED14
PAX7
PIGU
PPP2R5B
PTPMT1
SLC37A3
TADA2A
TAF1A
TMEM243
USP33
USP46
WDR90
ANO2
CDK5R2
LRFN4
TMEM179B
TREML2
ARIH1
BRI3BP
CRKL
CTBS
DNA2
EFTUD2
FBXL20
GLRX2
GRWD1
HGS
KBTBD8
KMT5B
MAN1A2
NAA38
NRM
PRPH
SEMA4B
SETD2
SPCS3
TNFSF13B
GTF2I
H2AC6
HLA-B
PIK3C2A
CFL2
KHSRP
PSMD2

RAB32
RUVBL2
SRP72
CHRNA7
ARFGAP1
ARFGEF3
CLASP1
CNPY2
DENR
DPY19L1
EMCN
FANCL
GTPBP1
MAPK15
MMACHC
NXPE3
RPL7L1
SCEL
SH3GL2
SHC4
TEAD2
TFB2M
USP43
ARHGDIG
CASTOR1
DHRS13
GP2
GTF3C3
MCM3AP
MEAF6
POC5
POP4
TAF1B
TBC1D10C
TBC1D19
UBE2D4
WFIKKN1
ZSCAN12
AKR1C18
ANKRD24
ARHGAP6
ARMC8
BRF2
BRWD3
CGGBP1
CLEC5A
DRAM2
EDEM2
FKRP
GRAMD4
HIRA

MCMBP
METTL21A
MIR125A
NSG2
NSUN5
RILP
SERGEF
SMAP2
SNX12
SOX3
SYT8
TICAM2
TSHZ3
ZIC3
EIF6
LNPEP
TBXA2R
ASIC1
BAZ2B
COL10A1
FSTL3
GATAD2A
NPC1L1
PRKG2
SEMA4G
SF3A1
SSR4
TANC2
TBCD
CRH
HRH1
DCTPP1
SKP1
EMILIN3
PLK5
RAB40C
SPRR2D
CCR4
DGKI
DVL3
FAM167A
FNTB
ITM2C
ITPRIPL2
LDLRAP1
LRRC8A
MARF1
METRN
PARVB
PHPT1
PKNOX2

POLR1A
PRPF38B
PRSS1
THOC1
TIMM44
TRIM29
TSPAN1
XYLT1
EEF1D
GABRB1
GSK3A
IPO5
AMY2B
CDC42EP1
CLCA1
CSDE1
ECPAS
ERAP1
FBXO9
KIF21B
MSRB3
MYL6
NAGA
RRP1B
SLC29A3
STIM1
STK38L
WNT7B
CD101
SKIC2
ANKZF1
ARHGEF5
ARMCX1
ATG13
COG3
CRACR2B
ENOPH1
ENSA
FGF11
GIP
GRID1
KCNC4
LIN37
OTUD6B
PLBD2
RABEP2
SLC37A1
SLCO5A1
SPATA17
TRIM33
ZNHIT6

IFFO1
IRF2BP1
KATNB1
KPNA5
LIMD1
MRPS9
RPH3A
TAF1C
UGT2B15
ATP6
KDM3A
ADCY4
ARPC5L
ATP8B2
CCDC77
CPA1
DCAF8
DHX37
DRD5
EFNB3
EIF2B5
GATA1
HEATR3
HINT2
IBTK
KIF2A
MEN1
MTM1
NENF
PCGF5
PHC3
RASSF3
SCFD1
SLC10A6
SLC25A45
SYTL5
UQCR10
VAMP4
WASF3
XPO7
CH25H
EPPK1
JMJD1C
NKD1
PHF19
ZC3HAV1
BAZ1B
CACNB2
KIF21A
NTRK3
NUCB1

OSBPL8
SCN4B
SUZ12
ZC3H6
ARL4D
A1BG
AMN
ANAPC4
ARL6IP4
BCS1L
CNPY3
DENND2A
DET1
DNAH5
FAHD2A
FAM135A
FAM184A
L3HYPDH
LMF1
LRRCC1
MGME1
MICALL2
MUP1
MYDGF
NRROS
ORC2
PAPPA2
PDZD8
PRSS36
SCAF1
SMG6
SMYD5
THOC3
TRAPPC3
UNC5CL
USP45
ATP5MC2
BMERB1
CCDC107
CCDC137
CSNK1G2
DGUOK
FLRT3
LRRC49
POP7
RNF216
RSAD1
SPRYD4
TSPYL1
USP42
ZC3H14

ARHGAP9
ATXN10
CAB39L
CORO2B
DDX42
ESF1
EXPH5
FAHD1
FHDC1
GAPVD1
HOOK2
KIF26B
LIPH
LRRC17
MAML2
TSPAN2
UTP6
VPS37A
ZFX
CDC37
CLN8
CPNE3
DYNC1LI2
GJA5
INSL3
KCNH1
KPNA1
KRT80
TSC22D2
NUP210
PLAC8L1
MTF1
MYO5B
NR0B1
RTP4
TNFSF4
CLDN6
DDHD2
GLYAT
GOSR1
HMBOX1
ICOSLG
IFT25
IFT81
ITFG2
MAP1S
MRPS16
NDFIP2
PPP4R4
SLC17A9
TAB1

TERF2
UNC13A
VAT1L
WDR44
CCK
OXT
CALB2
TG
KDELR2
MBOAT1
OBSL1
PEG3
RAB11FIP4
TNFSF12
ADGRF5
ARGLU1
ARHGAP15
BATF
BRWD1
COQ7
CTNND2
DDX20
FASTKD2
FRYL
GLIS3
METTL14
NR2C2
PARD3B
PHF14
POLR1D
RAG1
RALBP1
SIX4
SLC30A5
SMARCB1
STK3
TNKS2
TOM1
ABCF3
ACVR2A
ARL6
ARMC6
BMP8A
C3ORF52
CAPN9
CFAP53
FGF6
GPN1
H3F3A
KATNAL2
KCNQ4

LSG1
MSTO1
MUG2
NCDN
NRBP1
OLR59
PKD2L1
RNF5
SEC14L3
TMEM127
TMEM258
TMPRSS9
TRMT5
UPF3A
ZNF697
NRGN
RECQL4
SRGAP3
HTR1B
RYR2
SLC25A37
CHRNB2
CYP19A1B
EPM2AIP1
FBXW7
FHOD3
NSUN2
PARPBP
PLLP
PSMD3
RPS29
SDC3
WASF2
WDR6
ACRBP
AGPAT1
B3GAT1
CENPX
CEP41
CPNE5
FICD
HRC
HS3ST3A1
JOSD1
KIF13B
LGALS7
MCTP2
PHKA1
POFUT2
POMGNT2
RAB12

RTEL1
RUNDC3B
STARD3NL
TMEM40
USF2
USP39
VILL
WDR47
AHI1
ASTN2
CDC37L1
CDC5L
CIB1
CTNNA2
ETV3
HSF2
LSM5
MOB3B
NLRX1
PAIP1
PCYOX1L
PLCXD2
RIMS3
ROR2
SHC3
DCC
GLRX3
MARS1
PPP3R1
SEMA3A
PLCB4
DRD2
KRT25
PAX4
PPFIA2
ACTR1B
ANAPC7
DGCR6
FBRSL1
GSC
HAGHL
HSD3B
IFNLR1
LGI3
MFSD12
MRPL53
NAA30
NR6A1
NTAN1
OSCAR
PHF20

PPP1R21
SAV1
TMEM168
TMEM41A
ZNF704
FIBIN
MYCL
PPP1CC
RAB11FIP1
RAB5A
RPL23A
SH3PXD2B
TMC6
TNFRSF8
TRIM63
ARRB2
DCST1
KCNV1
KRT7
MED11
SECTM1B
TRIM34
H1-2
OSM
ABTB1
ATOSB
BET1
CACNA1S
CXCR5
DCTN4
DDX52
DGKB
DMPK
DNMBP
GABPA
H3C4
HCN2
HLA-DMA
IFT27
KCMF1
KCNJ16
KIFC2
LSM3
MIR221
N4BP2
NOL9
NUDT16L1
NUP37
PEX3
RNFT2
SERPINB6

SLC12A1
SLITRK5
STOML2
TMEM263
TMEM53
TMT1B
UGT8
UNC5D
UPK1A
VPS41
VPS45
XPNPEP3
BTNL8
CER1
WDR93
PRKG1
KCNQ2
MIPEP
NPM3
CACNA2D1
BEAN1
CIAO2A
COG6
CYP4A31
CYSLTR2
DALRD3
DIPK1B
DUSP13B
FUNDC2
GRIPAP1
H4C14
HABP4
KBTBD7
LRWD1
LY6G6C
MED15
NGLY1
NODAL
NXPH1
PDCL
PIP4P1
PMEL
RHBDD3
SLC6A18
SPRR1B
SPX
TAF12
THOC7
TMCO4
VEZT
WDFY2

ZFYVE27
ABLIM2
CIAPIN1
FAF1
FILIP1
FLII
GAL3ST1
IGSF9
NDUFC2
PGAM5
PHOSPHO1
SCN1A
TTC9
WDR46
ACOT11
BNIP1
CCDC141
COA7
DPY19L3
EXOSC6
GCC2
IL12RB1
KANK3
KHNYN
MED10
MED12L
MIR200C
NAIP
OSBPL2
PPP4R3B
RABGAP1
SAP30L
SLF1
SYNC
TRIM8
TSPAN9
ZC3H7B
CGA
CNP
RPL7
ANKRD23
SLC24A5
SPRR2A
ZNF32
CCL12
CDHR2
COL13A1
FAM89B
H2BC15
HHATL
LMOD3

MYCBPAP
NAT9
NT5M
PADI1
PNO1
SLC44A2
TRIM72
WDR43
ANO3
ATP6V0A2
C1GALT1
CCDC69
CSNK1G1
DYNC1I1
ELOB
FCGBP
HERC2
PAX5
PPIG
SIPA1L3
TLK1
TRIM36
TRPC6
AMER1
BRINP2
CCDC59
CEP250
COPS8
CTIF
CYP2C19
DMAP1
GSTCD
H3-4
HECTD4
IQSEC3
LZTR1
MIR34C
PICK1
PJA1
RNF180
RPRD2
SGCE
SHISA4
SPOCD1
STARD7
STRN4
AFF3
CD109
CPOX
DLG2
IFI47

IL3
KCNK5
MED13L
NEUROD1
RAI1
SLC20A2
VAPB
WDR77
ACER3
ANKRD29
B3GNT7
CAMSAP2
CEBPE
CLCA2
ELANE
KCNN3
MKRN1
MRPL40
NIPAL1
ORAI2
OSTF1
PFDN5
POLR3D
PPP4R1
PSMF1
RNF114
SEMA4C
SPTBN4
SUMF2
URB2
ZDHHC13
AMH
GNAQ
CES3A
ACMSD
CHRNE
CYP46A1
DNAJC15
GABRP
LSM6
MALL
OAS1A
POC1A
SLC16A14
STAG1
TEAD4
C4BP
CAVIN1
CEP112
DYNC2I1
ENTR1

KCND1
KLHL32
MIR30D
MIR30E
RASGEF1C
RGS13
ULK3
YIPF2
ZSCAN2
CD99L2
EXOSC2
HEPH
MCU
MIS18A
EBF4
KLK1B4
LY6G
MIR148A
SERPINA3G
UGT2B36
ZNF580
ANKRD26
ATP6V1C2
CCDC85A
DNASE2
DYRK1B
ELOA
FGF15
GNPDA2
GPR88
MNT
PLA2G12B
PLEKHG4
POP5
RBM4
RNF150
SDCCAG8
SEC11C
SF3A3
SLC16A13
SSBP4
STON1
SV2B
TSGA10
UBLCP1
ABHD16A
ADPRM
CABP1
CASQ1
DNAJC4
EDRF1

EMC3
ENOSF1
GCG
LARS2
LSM14A
LSMEM1
MIR23A
MSTN
MTO1
NCKIPSD
NCOR2
NDP
PGC
PIKFYVE
SHE
SYF2
UQCC1
VPS11
GRIN2B
ARAP1
CCL1
CENPV
CLCN7
CTRB1
ERC2
FAM149A
GJB3
MAP3K2
MARK1
MSH3
N4BP1
NPEPL1
NTM
SLC17A1
SNRPN
SRSF4
SSR2
USP24
GALNT6
IMPA1
MEX3B
MYOG
PLD2
TXNDC5
APBA1
ATG4D
CCDC47
CFHR1
CSRNP2
CTSO
CYP2J4

GOLGA7
HIF1AN
HS6ST3
IFT20
INSC
IRAK1BP1
KCNJ9
LIX1
MIR126
NBEAL2
NIF3L1
NTPCR
PHC1
RALGPS1
RESP18
RINT1
RIPPLY3
SLC17A3
SLC35A3
SNX7
SPATA18
SPATA7
TBC1D30
TP53BP2
UBAP2
UBE2D1
WDR7
WFDC18
WNT16
ZNF22
ADCK5
AP4E1
ARFRP1
BLOC1S1
DDN
ENDOU
FBXL6
FN3KRP
HEATR6
LHX4
LRRC3B
LRRC42
MRS2
MS4A6A
MYL3
PISD
RNF10
RNF11
SLC35F3
SLC9A9
SMUG1

SYDE2
VAMP3
WWC3
GPSM1
IRX5
OTX2
PRRC2A
RPL29
SPART
UBFD1
UHMK1
CNTD1
PDE10A
UGT1A3
APBB1
BOLA1
C1RL
CDK16
CLDND1
D2HGDH
DPF1
EPC1
EPHB1
GPR160
MCRIP2
MED21
MGAT4A
MINDY1
MOCS2
MRM3
MRPS30
MS4A7
NKX2-5
POLR2L
PPFIA3
PRR15
SBSPON
SFT2D2
SLC9A7
TM6SF2
TRIM26
UBAP1
EPHA3
GDI1
TIGAR
VARS1
AVP
ARHGAP23
EFNA3
IFI16
NAA50

RNF43
SLC2A8
VPS13A
GRIN2C
AP3S2
BCDIN3D
C1QL1
CCDC152
CDK5RAP1
CHMP2A
COMMD2
CYB561D2
DIRAS2
ENTPD8
FBXO25
FCRL2
FMNL1
FZD9
GLB1L2
HOXA3
IL17RE
KLHL17
KLHL26
MIR183
MIR99A
NAA40
NDUFAF2
NEU3
NEURL2
NMNAT3
ORMDL1
PPCDC
PRDM8
PRELID3B
PTPRCAP
SCN2B
SERF2
SOX1
SPOPL
STX18
SYAP1
TMEM131L
TUBGCP6
TXNDC12
TXNDC9
UBE2Q1
USF1
WASHC5
XKRX
ZBTB41
ZNF473

ZSCAN21
DOCK9
FTO
RPL11
RPS26
TBC1D2
BAG5
CPNE1
CSNK1G3
CYTH4
ELMO2
ENY2
FKBP2
GLYCTK
GNB4
GRHL3
HOOK1
ING1
INTS3
ITPKC
MEIG1
MIR141
MRPS17
MRPS18C
NELL1
OTUD4
PLA2G15
REXO4
RIMS2
SH3RF3
TMEM109
URI1
ARL8B
CBL
CCDC88C
DUSP7
EFHC1
GDE1
HOMER1
KLHL29
PDHB
RBM8A
SEC13
SERPINA3
ST3GAL3
TNXB
ZBTB21
ADAMTS16
ASB10
COXFA4L2
FAM199X

GSG1
NUP42
RELCH
SELENOO
SNURF
CHRM2
ADNP
KCNJ11
ABHD17B
ASB7
B3GAT2
BCAN
CHST7
COQ8B
CSTF2T
FAM169A
FTSJ1
GOLPH3L
HCCS
HIC2
LMTK2
LTB4R
MB21D2
MIR150
MIR93
MRPL52
PQBP1
PRR5
SHPRH
SLAMF1
SNRNP35
SNX21
TAFA5
TIMM8A1
UTP25
CACNA1D
DRD1
ND1
PAPPA
TNIK
WFDC12
ZNF710
ANAPC13
ARF2
EPYC
FAM3D
IFITM6
MIR146B
MIR203
NKX6-1
P2RY13

PCGF6
PLAC9
PRRG2
RABIF
REM1
RHPN1
RNF135
SCYL3
SIX2
TANGO6
TLE6
TRIM41
TTLL11
VPS37C
ACVR2B
ANO6
BEX2
EPHA7
FKTN
HAPLN1
KDM6A
MIB2
PNKP
RASGEF1B
SREK1
UNKL
VWA5A
BET1L
CACHD1
CYP4F2
DAGLA
DENND5B
DPP6
HK3
KIRREL3
L2HGDH
M6PR
MMS22L
MOSPD1
NDUFA6
PLEKHM1
RDM1
RHEBL1
SEMA3D
SF3B2
STOX2
TAF7
TOMM34
TSFM
UBE2V2
ZC3H11A

NUP93
ESRRG
ADAMTS14
AGBL2
AP1G2
ARHGEF10L
ATL3
AVIL
BAHCC1
C1QTNF6
CDC42BPG
CDK11B
CREB3L4
DCPS
GCC1
GIN1
GRIK5
GUCD1
H1-10
HOXB7
IDS
ING3
KCNK2
LYRM7
MDFI
MRPL34
MYBPH
NAA80
NOP16
OPRL1
PITPNM2
PPP1R16B
PPWD1
PRKRA
PTRH2
PYROXD1
RAD17
RASL12
RC3H1
RNASE6
SLTM
TMED9
TMEM160
UFM1
UGT2B35
ZMYND10
ZNF827
IYD
ANGEL1
ARPC3
CAMSAP1

CBLC
CDK13
FAM47E
FAU
GP1BB
HELZ
HIPK1
HYAL2
IL12RB2
IP6K1
ITGBL1
MAPK8IP1
MROH6
NXPE2
PHF11
PIP4K2B
POU2AF1
RAP2A
RFWD3
RNF39
RTL8C
SELENOK
SMN1
VASH2
XCL1
XIRP1
MT1G
SLCO1A2
ZBTB10
CPLANE1
DHX32
DMRT2
DUSP15
GLIS1
GNGT1
LRRC75A
PNLIPRP2
PODN
SERPINB6A
SIGLEC5
SPINK4
TARBP1
TMEM167A
UBXN2A
DOCK7
DPP9
ELOC
EPB41L4B
GTF2F1
ND2
SLC13A4

APOBEC3F
SECTM1A
AHNAK2
CCDC86
DHODH
ETV6
PPP2R5C
UBR4
AOC2
BMP3
CEACAM6
CECR2
CLIC2
CMAH
COX10
CPTP
CRB2
FCRLA
FRAT2
GPR132
HEATR5A
HOXB2
HYDIN
IL3RA
ISL2
KLHL18
LMBRD2
MCTS1
MTHFS
NEUROG2
P2RY10
PLEKHA4
POU4F2
RASIP1
RGS14
RPP25
SRFBP1
STX8
UBE2G1
UBXN7
WDR81
ZBTB26
ZNRF3
AKAP7
APC2
ASIC2
B9D1
CAPZA2
CNOT6L
H2-AB1
MYO9B

NPHP1
NUBP2
OPHN1
PAQR8
PLEKHA7
PLPBP
VWCE
NF1
SP7
