# Supplementary material for: Bioinformatics Profiling of a Paeoniflorin-Associated “Macrophage–Lactylation” Axis in Hepatocellular Carcinoma: An LDHA-Based Prognostic Model and Structural Computational Evidence
Source: Int J Mol Sci. 2026 Mar 9;27(5):2495. doi: 10.3390/ijms27052495 (PMC12986180; doi:10.3390/ijms27052495)
Supplement: Supplementary file 1 [file ijms-27-02495-s001.zip › Supplementary File/ijms-4165836-Table Supplementary1-4.zip!/ijms-4165836-Table Supplementary1-4/ijms-4165836-Supplementary Table3.pdf]

HMGB1
WAS
TP53
SOD1
TFEB
LRP1
VIM
SIRT1
EP300
RB1
HDAC1
ALB
RUNX2
HDAC2
PARP1
CALR
HDAC3
GAPDH
LGALS1
ATM
CREBBP
G6PD
NPM1
IKZF1
MBP
MAP2K4
PRKDC
HCFC1
YAP1
TKT
AARS1
APOC2
NCL
MSN
LSP1
TMSB4X
NR4A3
FASN
LRPPRC
STK11
PRDX1
ADAR
CALM1
NBN
LCP1
PPIA
CGAS
GFAP
KAT5
AARS2

IRF2BP2
PDHA1
BRD4
PIK3C3
PFN1
ILF3
DHX9
IFI16
ZNFX1
SFPQ
ALDOB
TCOF1
LDHA
H3-3B
HMGA1
S100A4
HNRNPD
HNRNPA1
H3C1
YY1
NCOA4
MKI67
PABPC1
HEXIM1
ADNP
PTMA
EEF1A1
METTL3
HNRNPM
HNRNPU
KHDRBS1
GPR37
PTBP1
MECP2
ENO1
FABP5
RPL29
H4C16
SIRT3
DDX5
ILF2
H2BC21
JMJD1C
HNRNPL
H2BC12L
BCLAF1
HNRNPH1
SLC16A4
RBM17
IK

H3-3A
PGK1
TMPO
ULK1
RPS27A
RPS11
RARG
HDGF
RPL13
H2BC11
RANBP2
RACGAP1
SRRM2
H4C4
DDX39A
RSL1D1
SRP14
ACIN1
SSB
HSPE1
MNDA
CHD4
HNRNPK
PSMA7
HMGN2
NONO
HNRNPF
KHSRP
CNN2
DDX21
S100A11
PAK2
ALDOA
UPF1
RAN
RAD50
DDX39B
NOP2
NUDT21
ALYREF
LDHB
CBX3
MDC1
CCNA2
SAFB
DDX17
BZW1
ZC3H4
PRCC
SH3GL1

HNRNPC
ARID1A
POLDIP3
SIRT2
TRIM28
H4C1
ZYX
NUP133
H4C3
H4C5
RPL14
SUMO2
MAGOHB
H2BC12
EIF4G1
KRT1
H4C14
GTF2I
RBMX
ALDH1A1
OSBP2
PRPF6
THRAP3
H2BC26
TERF2
RECQL
H4C15
EEF2
NHLRC2
EIF4G2
ARID3B
PPP1CC
ACAT2
NREP
RRP1B
H2BC4
CALML5
H2AZ1
H2BC5
H3C12
H3C2
H4C6
H2BC3
H3C13
H2BC14
H4C11
H4C8
H4C9
H3C10
H3C14

H3C3
H3C4
H3C6
H3C8
H4C2
H2BC1
H2BC13
H2BC15
H2BC9
H3C11
H3C7
H4C12
H4C13
H2BC17
H2BC10
H2BC18
H2BC6
H3C15
H2BC7
H2BC8
RPL22
