# Supplementary material for: Bioinformatics Profiling of a Paeoniflorin-Associated “Macrophage–Lactylation” Axis in Hepatocellular Carcinoma: An LDHA-Based Prognostic Model and Structural Computational Evidence
Source: Int J Mol Sci. 2026 Mar 9;27(5):2495. doi: 10.3390/ijms27052495 (PMC12986180; doi:10.3390/ijms27052495)
Supplement: Supplementary file 1 [file ijms-27-02495-s001.zip › Supplementary File/ijms-4165836-Table Supplementary1-4.zip!/ijms-4165836-Table Supplementary1-4/ijms-4165836-Supplementary Table4.pdf]

SOD1
LDHA
ALB
NPM1
SIRT1
GFAP
STK11
HNRNPU
